# *Oxybaphus himalaicus* Mitigates Lipopolysaccharide-Induced Acute Kidney Injury by Inhibiting TLR4/MD2 Complex Formation

**DOI:** 10.3390/antiox11122307

**Published:** 2022-11-22

**Authors:** Honghong Zhan, Qingxiu Pu, Xiaoliang Long, Wei Lu, Guowei Wang, Fancheng Meng, Zhihua Liao, Xiaozhong Lan, Min Chen

**Affiliations:** 1 Key Laboratory of Luminescence Analysis and Molecular Sensing (Southwest University), Ministry of Education; College of Pharmaceutical Sciences, Southwest University, Chongqing 400715, China; 2 School of Life Sciences, Integrative Science Center of Germplasm Creation in Western China (CHONGQING) Science City and Southwest University, The Provincial and Ministerial Co-Founded Collaborative Innovation Center for R&D in Tibet Characteristic Agricultural and Animal Husbandry Resources, TAAHC-SWU Medicinal Plant Joint R&D Centre, Southwest University, Chongqing 400715, China; 3 TAAHC-SWU Medicinal Plant R&D Center, Tibet Agricultural and Animal Husbandry University, Nyingchi 860000, China

**Keywords:** *Oxybaphus himalaicus*, TLR4/MD2, NADPH oxidase 2, inflammation, reactive oxygen species, acute kidney injury

## Abstract

Acute kidney injury (AKI) is described as the abrupt decrease in kidney function always accompanied by inflammation. The roots of *Oxybaphus himalaicus* Edgew. have long been used in Tibetan folk medicine for the treatment of nephritis. Nevertheless, modern pharmacological studies, especially about the underlying mechanism of *O. himalaicus* medications, are still lacking. Here, in lipopolysaccharide (LPS)-induced RAW264.7 macrophages, the *O. himalaicus* extract (OE) showed significant anti-inflammatory activity with the dose dependently reducing the LPS-stimulated release of nitric oxide and the mRNA level and protein expression of inflammatory cytokines and reversed the activation of nuclear factor kappa B (NF-κB). Co-immunoprecipitation assay indicated that OE inhibited Toll-like receptor 4/myeloid differentiation factor 2 (TLR4/MD2) complex formation and further suppressed both myeloid differentiation factor 88 (MyD88)-dependent and TIR-domain-containing adapter-inducing interferon-β (TRIF)-dependent cascades activation. In addition, OE could restrain NADPH oxidase 2 (NOX2) endocytosis by blocking TLR4/MD2 complex formation to prevent reactive oxygen species production. In LPS-induced AKI mice, OE treatment mitigated renal injury and inflammatory infiltration by inhibiting TLR4/MD2 complex formation. UPLC-MS/MS analysis tentatively identified 41 components in OE. Our results indicated that OE presented significant anti-inflammatory activity by inhibiting TLR4/MD2 complex formation, which alleviated LPS-induced AKI in mice.

## 1. Introduction

Acute kidney injury (AKI) refers to a dramatic reduction of kidney function, while long-term AKI leads to irreversible chronic kidney disease (CKD) or end-stage renal disease [1]. There is an increasing risk of mortality and morbidity associated with AKI, in which an inflammatory response commonly exists [2]. Consequently, inhibition of inflammation plays a significant role in treating AKI.

Macrophages play a critical role in generating inflammation. When Toll-like receptors (TLRs) are activated, macrophages initiate inflammatory responses by activating specific transcriptional cascades [3]. TLRs activation in macrophages has a significant impact on recognizing extracellular pathogens and activating an innate immune response. Toll-like receptor 4 (TLR4) is the only known TLRs to activate genes that encode inflammatory molecules via both myeloid differentiation factor 88, (MyD88)-dependent, and TIR-domain-containing adapter-inducing interferon-β, (TRIF)-dependent cascades, especially by lipopolysaccharide (LPS), which plays a significant role in triggering cellular inflammation [4,5]. Briefly, LPS binds to serum lipopolysaccharide-bind protein, by which CD14 captured LPS and then transferred to the Toll-like receptor 4/myeloid differentiation factor 2 (TLR4/MD2) complex on the cell membrane. Subsequently, activated TLR4/MD2 complex dimerized to regulate downstream proteins, such as nuclear factor-κB (NF-κB) and mitogen-activated protein kinase (MAPK) signaling pathways, by TIRAP/MyD88 and TRAM/TRIF-dependent cascades, to initiate an inflammatory response [6]. Furthermore, reactive oxygen species (ROS) are largely generated in tissue-infiltrated macrophages during inflammation. Nicotinamide adenine dinucleotide phosphate (NADPH) oxidase 2 (NOX2), also known as gp91phox, is a phagocyte-specific NADPH oxidase that tremendously contributes to ROS synthesis in macrophages [7]. Therefore, regulating TLR4 and NOX2 activation in strategic ways to alleviate an inflammatory response.

The root of *Oxybaphus himalaicus* Edgew., also referred to as *Mirabilis himalaica* (Edgew.) Heim. (Nyctaginaceae), is a Tibetan medicine approved by the Pharmacopoeia Committee of the Ministry of Health of the People’s Republic of China. It has been used for the treatment of nephritis, edema, arthralgia, and impotence [8,9]. However, modern pharmacological studies about the traditional effect of *O. himalaicus* are still scarce. One study reported that benzofuran ε-caprolactam glucosides, amides, and phenylpropanoid derivatives from *O. himalaicus* showed anti-inflammatory activity [10] that is moderate with a single compound. Considering that inflammation frequently occurs in AKI and the traditional effect of *O. himalaicus* on renal diseases, it is necessary to systematically explore the anti-inflammatory activity and its underlying mechanisms. In this study, we identified the most potent anti-inflammatory parts of the *O. himalaicus* extract (OE) and explored how they function in vitro. Subsequently, the anti-inflammatory effect and the underlying mechanism of OE were validated in LPS-induced AKI mice.

## 2. Materials and Methods

### 2.1. Reagents

Lipopolysaccharide (LPS), L-canavanine (L-cana), Protein A + G Agarose, and 2-(4-Amidinophenyl)-6-indolecarbamidine dihydrochloride (DAPI) were obtained from Beyotime (Shanghai, China). Dynasore (Dyn) was purchased from MedChemExpress (Monmouth Junction, NJ, USA). Dulbecco’s modified Eagle’s medium (DMEM) and fetal bovine serum (FBS) were acquired from Gibco (Carlsbad, CA, USA). The primary antibody targeting TLR4 (19811-1-AP), MyD88 (67969-1-Ig), iNOS (22226-1-AP), COX-2 (27308-1-AP), TNF-α (17590-1-AP), IL-6 (21865-1-AP), p65 (66535-1-Ig), EEA1 (68065-1-Ig), p-JNK (80024-1-RR), and JNK (24164-1-AP) were obtained from Proteintech (Wuhan, China). IκΒ (AF1282), p-p65 (AF5881), TRIF (AF8238), NOX2 (AF7596) and β-actin (AF0003) were purchased from Beyotime (Shanghai, China), MD2 (YN2063) was acquired from Immunoway (Plano, TX, USA), and p-ERK (bs-3016R), ERK (bsm-52259R), p-p38 (bs-0636R) and p38 (bs-0637R) were obtained from Bioss Antibodies (Beijing, China). HRP-conjugated anti-Heavy Chain of Rabbit IgG (SA00001-7H) was purchased from Proteintech (Wuhan, China). HRP-conjugated anti-rabbit IgG (A0208) and anti-mouse IgG (A0216), Alexa Fluor 488-conjugated (A0428) and FITC-conjugated (A0568) anti-mouse IgG, and Cy3 conjugated anti-rabbit IgG (A0516) were acquired from Beyotime (Shanghai, China). Rabbit IgG (GB111738) was purchased from Servicebio (Wuhan, China). TLR4 siRNA (siTLR4) and negative control siRNA (siNC) were obtained from Tsingke Biotechnology (Beijing, China). Lipofectamine 3000 transfection reagent was purchased from Invitrogen (Carlsbad, CA, USA).

### 2.2. Extraction of O. himalaicus

The roots of *O. himalaicus* were collected from Nyingchi County, Tibet Autonomous Region, People’s Republic of China, in March 2021. Samples were identified by Professor Xiaozhong Lan of Tibet Agricultural and Animal Husbandry University. A voucher specimen (No. 2021-CM-002) was deposited at the College of Pharmaceutical Sciences, Southwest University, Chongqing, China.

The dried roots of *O. himalaicus* (16.50 kg) were crushed and extracted with 95% ethanol three times. Then, the total 95% ethanol extract (TE, 1.10 kg) was freeze-dried and dissolved in water by ultrasonics. The solution was fractionated on D101 macroporous resin with 0%, 40%, 60%, 80%, and 95% ethanol, respectively, to obtain fractions A-E for further anti-inflammatory activity screening. The flow chart of the extraction and purification of OE is presented in Figure S1. The most powerful anti-inflammatory fraction, B (OE), was obtained for further analysis.

### 2.3. Cell Culture

RAW264.7 macrophages were obtained from the Cell Bank of the Chinese Academy of Sciences (Shanghai, China). Cells were cultured in DMEM with 10% FBS in a humidified incubator with 5% CO2 at 37 °C. Additionally, for contamination prevention, 1% penicillin-streptomycin solution was added to the culture medium.

### 2.4. MTT Assay

RAW264.7 macrophages were seeded into 96-well plates at a density of 1 × 10^4^ cells per well and cultured for 24 h. Then, cells were treated with different concentrations of fraction A-E isolated *O. himalaicus* and cultured for 24 h. Cell viability was detected by MTT assay according to our previous study [11].

### 2.5. Nitric Oxide Measurement

RAW264.7 macrophages were seeded into 48-well plates at a density of 1.5 × 10^5^ cells per well and cultured for 24 h. Afterward, cells were pretreated with the indicated concentration of fraction A-E for 3 h. Then, LPS (1 μg/mL) was added and cultured for 24 h. The medium was harvested, and nitrite was measured by an NO detection kit (Beyotime, Shanghai, China) according to the manufacturer’s instructions. L-canavanine (L-cana), an inhibitor of iNOS, was used as a positive control.

### 2.6. Reactive Oxygen Species Detection

RAW264.7 macrophages were seeded into 6-well plates at a density of 1.5 × 10^6^ cells per well and cultured for 24 h. Subsequently, cells were pretreated with OE (20, 40, 80 μg/mL) for 3 h. Whereafter, LPS (1 μg/mL) was added and cultured for 6 h. Then, a reactive oxygen species (ROS) level was measured by a ROS detection kit (Beyotime, Shanghai, China) according to the manufacturer’s indication via flow cytometry (Becton, Dickinson & Company, Franklin Lakes, NJ, USA). Data were analyzed by FlowJo 10.8.1 software (Becton, Dickinson & Company, Franklin Lakes, NJ, USA).

### 2.7. Immunofluorescent Staining

RAW264.7 macrophages were seeded into 96-well plates at a density of 5 × 10^3^ cells per well and cultured for 24 h. Then, cells were pretreated with OE (80 μg/mL) for 3 h, and LPS (1 μg/mL) was subsequently added and cultured for the indicated time. Immunofluorescence staining was performed as per our previous report [12]. Images were captured at 400× magnification with confocal mode by Operetta CLS High Content Analysis System (PerkinElmer, Waltham, MA, USA).

### 2.8. SiRNA Transfection

RAW264.7 macrophages were transfected with 100 nM siNC or siTLR4 loaded with Lipofectamine 3000, according to the manufacturer’s instructions. Sequences (5′-3′) of siRNA are presented in Supplementary Materials (Table S1).

### 2.9. Animal Model and Design

C57BL/6J male mice, weighing 20 ± 2 g, were purchased from Hunan SJA Laboratory Animal Co., Ltd. (Hunan, China). The specific pathogen-free (SPF) conditions of controlled temperature (25 °C) and humidity (50%) were applied to the mice during the experiments. All analyses of the animals were approved by the Institution of Animal Care and Use Committee (IACUC) of Southwest University (IACUC-20220525-01).

Mice were randomly divided into normal (*n* = 16), low dose-OE (100 mg/kg, *n* = 8), medium dose-OE (200 mg/kg, *n* = 8), and high dose-OE groups (400 mg/kg, *n* = 8). OE was dissolved in 20% propylene glycol (PG). Before the experiment, mice were adapted to the environment for 7 days. Then, 100, 200, and 400 mg/kg of OE were administered to the mice in the low, medium, and high dose-OE groups by gavage for a consecutive 7 days, respectively, while an equivalent amount of 20% PG was intragastrically administered to the normal group. After administration for 7 days, the mice in the normal group were randomly divided into the control group (*n* = 8) and the LPS group (*n* = 8). LPS (10 mg/kg) dissolved in sterile Phosphate Buffer Saline (PBS) was intraperitoneally administered to the mice in the LPS and OE groups. Simultaneously, an equivalent volume of sterile PBS was intraperitoneally administered to the mice in the control group. Body weight was measured during the experiment. After fasting for 12 h, mice were anesthetized and sacrificed. Serum and kidney samples were collected for further analysis.

### 2.10. Serum Biochemical Analysis

Serum creatinine (CRE) and blood urea nitrogen (BUN) were measured by a CRE assay kit (Nanjing jiancheng, Nanjing, China) and a BUN assay kit (Nanjing Jiancheng, Nanjing, China), respectively, in accordance with the manufacturer’s instructions.

### 2.11. Histopathology and Immunochemistry

Mice kidney samples were fixed with 4% paraformaldehyde, and HE and immunochemical staining were performed as per our previous study [13]. In total, 10 fields from each group were captured at 200× magnification by fluorescent microscopy (Nikon, Tokyo, Japan). For immunochemical analysis, Image J software (National Institutes of Health, Bethesda, MO, USA, Bethesda, MD, USA) was applied to analyze the positive area in each field.

### 2.12. Dihydroethidium Staining

ROS generation in the kidney was detected by Dihydroethidium (DHE) staining. Kidneys were quick-frozen in liquid nitrogen, and 10 μm cryosections were obtained via freezing microtome (Thermo Scientific, Waltham, MA, USA). Then, the DHE staining solution (Sigma Aldrich, Saint Louis, MO, USA) was added to the marked area of the kidney and incubated at 37 °C for 30 min in a dark place. Afterward, DAPI was added for 10 min to counterstain the nucleus. Finally, slips were covered with an anti-fade mounting medium. 8 fields in each group were randomly selected and captured at 200× magnification via fluorescent microscopy. Image J software was applied to analyze the mean DHE fluorescent intensity.

### 2.13. Quantitative Real-Time PCR

The total RNA in RAW264.7 macrophages or kidney tissues was extracted by an RNA extraction kit (Beyotime, Shanghai, China). 1 μg total of RNA was used to perform reverse transcription to obtain cDNA via the FastKing-RT SuperMix reagent (Tiangen, Beijing, China). Quantitative real-time PCR was performed with the SYBR Green qPCR Master Mix (APExBIO, Houston, TX, USA). The mRNA level of each target gene was normalized to GAPDH for semi-quantitative analysis. Primer sequences (5′-3′) are presented in Supplementary Materials (Table S2).

### 2.14. Western Blotting

Membrane and cytoplasm proteins in RAW264.7 macrophages were isolated by means of a membrane and cytoplasm protein extraction kit (Beyotime, Shanghai, China). Total protein extraction in RAW264.7 macrophages or kidney tissues and immunoblotting were performed in accordance with the previous report [14]. Visualization of the membranes was achieved using a sensitive chemiluminescence regent (Proteintech, Wuhan, China). The protein expression level of each target protein was normalized to β-actin for semi-quantitative analysis. Immunoreactive bands in each membrane were analyzed by Image J software.

### 2.15. Co-Immunoprecipitation Assay

RAW264.7 macrophages or kidney tissues were lysed with a lysis buffer. A 50% protein A + G agarose was added into the lysate and incubated for 30 min. Precipitates were discarded after centrifugation at 2500 rpm for 5 min to avoid non-specific binding. Then, protein samples were incubated with an anti-TLR4 antibody overnight while normal rabbit IgG was used as a negative control. Subsequently, the protein A + G agarose was added to the mixture and incubated for another 3 h. After centrifuging and washing with ice-cold PBS 5 times, immune precipitates were acquired for further immunoblotting analysis.

### 2.16. Analysis of Components in OE

The analysis of the components in OE was carried out using the Ultimate 3000 Ultra Performance Liquid Chromatography system with the Q Exactive Orbitrap Liquid chromatography-Mass spectrometry (UPLC-MS/MS) (Thermo Scientific, Waltham, MA, USA) operated at 35 °C and equipped with a Hypersil GOLD C18 column (2.1 mm × 100 mm, 1.9 µL) (Thermo Scientific, Waltham, MA, USA). 100 μg/mL of OE was applied for the subsequent analysis. The component analysis was achieved using UPLC-MS/MS in both negative and positive modes, including full MS scans and data-dependent MS2 (ddMS2) scans. The obtained MS/MS spectrometry data was analyzed with Compound Discoverer 3.0 Software (Thermo Scientific, Waltham, MA, USA), and the results were matched in the mzCloud database (www.mzcloud.org (accessed on 12 September 2022)). Compounds with a mzCloud best match score greater than 85 points were taken into the following exploration.

### 2.17. Statistical Analysis

All data, represented as mean ± SD, were repeated at least 3 times independently. The statistical significance was analyzed with an unpaired Student’s *t*-test comparing the two groups and one-way analysis of variance (one-way-ANOVA), followed by Dunnett’s post hoc test for multiple comparisons, via GraphPad Prism 7.0 software (GraphPad Software, San Diego, CA, USA). Respectively, *p* < 0.05 and *p* < 0.01 were statistically significant and highly significant.

## 3. Results

### 3.1. OE Dose Dependently Alleviates LPS-Induced Inflammation in Macrophages

**Figure 1:**
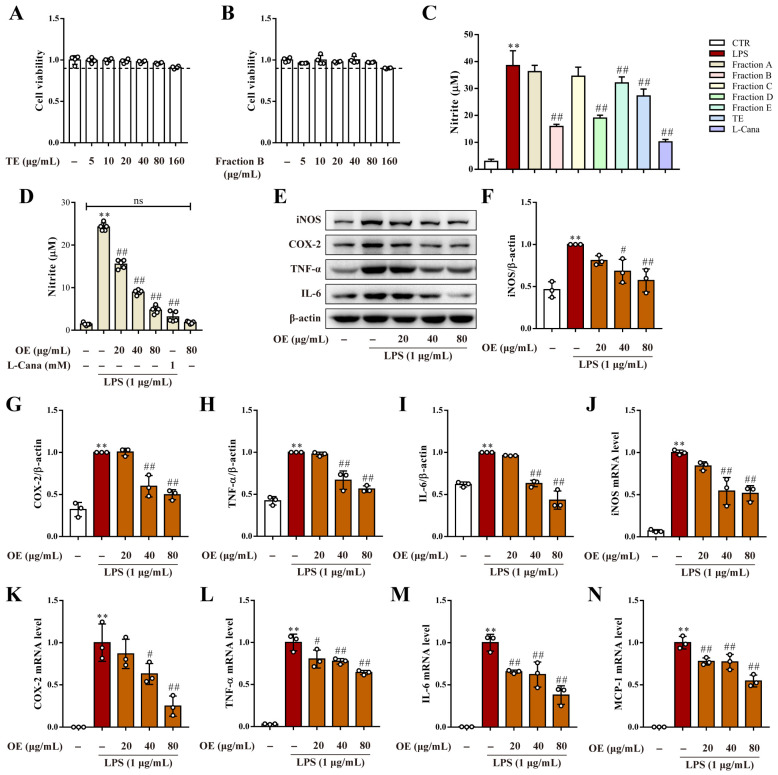
OE alleviated LPS-induced inflammation in macrophages. (**A**,**B**) Cell viability of TE (**A**) and fraction B (**B**) on RAW264.7 macrophages. (**C**,**D**) RAW264.7 macrophages were pretreated with the indicated concentration of various substances for 3 h followed by LPS (1 μg/mL) stimulation for 24 h. The medium was collected to determine the NO release level. (**E**–**I**) RAW264.7 macrophages were pretreated with OE (20, 40, 80 μg/mL) for 3 h followed by LPS (1 μg/mL) stimulation for 24 h. Total protein expression level of iNOS, COX-2, TNF-α, and IL-6 were detected by western blotting assay. (**J**–**N**) RAW264.7 macrophages were pretreated with OE (20, 40, 80 μg/mL) for 3 h followed by LPS (1 μg/mL) stimulation for 6 h. The mRNA level of iNOS (**J**), COX-2 (K), TNF-α (**L**), IL-6 (**M**), and MCP-1 (**N**) were determined by quantitative real time PCR analysis. CTR: control. Data presented as Means ± SD. ** *p* < 0.01 vs. control group; # *p* < 0.05, ## *p* < 0.01 vs. LPS group; ns, no significance.

Data revealed that the total 95% ethanol extract (TE) significantly reduced the LPS-induced NO release level in the culture medium of RAW264.7 macrophages without an effect on cell viability. When paralleled to the iNOS inhibitor L-canavanine (L-cana), subsequent screening showed fraction B most powerfully declined the NO release level with no cytotoxicity to RAW264.7 macrophages. A high concentration of other fractions exhibited moderate to no effect on NO release but simultaneously had distinct cytotoxicity (Figure 1A–C) (Figure S2). Therefore, fraction B was identified as the anti-inflammatory part of the *O. himalaicus* extract (OE). Further study showed that the OE dose dependently reduced the NO released in the medium on RAW264.7 macrophages (Figure 1D). Furthermore, the OE significantly declined in inflammation-related protein expression, including iNOS, COX-2, TNF-α, and IL-6 (Figure 1E–I). Moreover, LPS-induced high mRNA levels of iNOS, COX-2, TNF-α, IL-6, and MCP-1 were also reversed by OE (Figure 1J–N).

### 3.2. OE Restrained LPS-Indued NF-κB Activation in Macrophages

**Figure 2:**
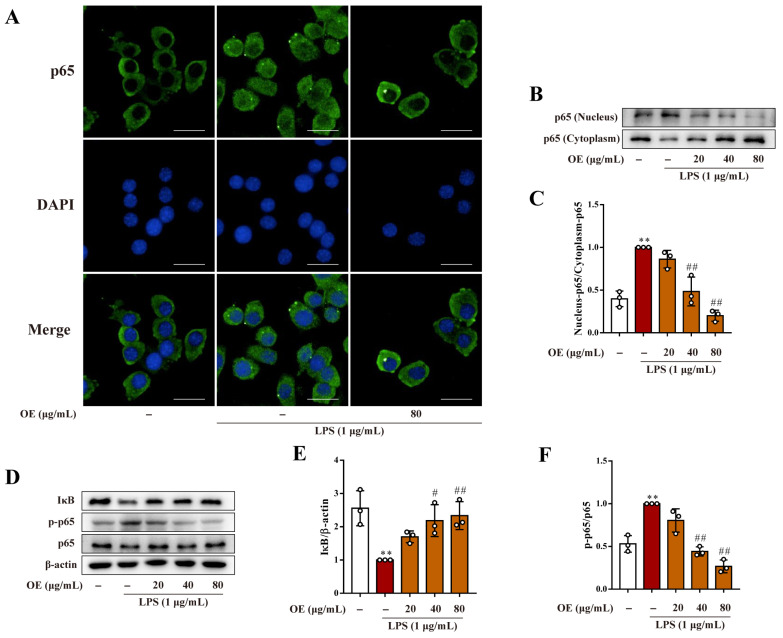
OE suppressed NF-κB activation in macrophages. (**A**) RAW264.7 macrophages were pretreated with OE (80 μg/mL) for 3 h followed by stimulation with LPS (1 μg/mL) for 2 h. p65 nuclear translocation was detected by immunofluorescent staining. Scale bars: 20 μm. (**B**–**F**) RAW264.7 macrophages were pretreated with OE (20, 40, 80 μg/mL) for 3 h followed by LPS (1 μg/mL) stimulation for 2 h. Nuclear and cytoplasmic protein expression of p65 (**B**,**C**) and the protein expression level of IκB, p-p65, and p65 (**D**–**F**) were determined by a western blotting assay. Data presented as Means ± SD., ** *p* < 0.01 vs. control group; # *p* < 0.05, ## *p* < 0.01 vs. LPS group; ns, no significance.

NF-κB activation is responsible for regulating many proinflammatory genes in an inflammatory response [15]. OE significantly suppressed the LPS-stimulated nuclear translocation of the p65 subunit of NF-κB in macrophages (Figure 2A–C). Moreover, OE repressed the degradation of the inhibitor of NF-κB (IκB) and the phosphorylation of p65, both of which play critical roles in NF-κB activation (Figure 2D–F). Collectively, OE remarkably restrained LPS-stimulated NF-κB activation in macrophages.

### 3.3. OE Inhibited TLR4/MD2 Complex Formation in Macrophages

**Figure 3:**
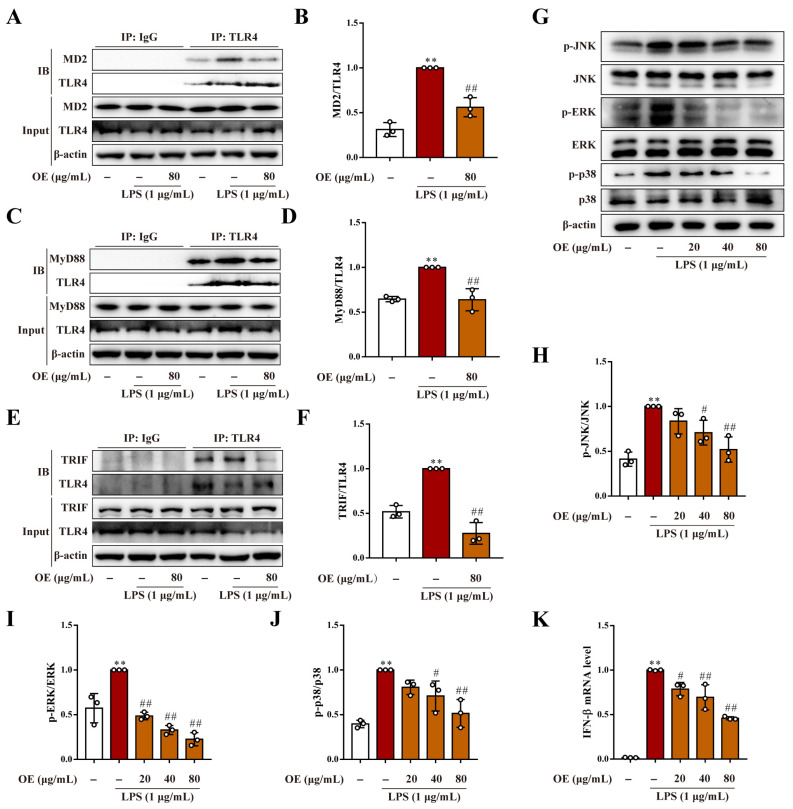
OE inhibited TLR4/MD2 complex formation. (**A**–**F**) RAW264.7 macrophages were pretreated with OE (80 μg/mL) for 3 h followed by stimulation with LPS (1 μg/mL) for 2 h. Interaction between TLR4/MD2 (**A**,**B**), TLR4/MyD88 (**C**,**D**), and TLR4/TRIF (**E**,**F**) were determined by a co-immunoprecipitation assay. (**G**–**J**) RAW264.7 macrophages were pretreated with OE (20, 40, 80 μg/mL) for 3 h followed by stimulation with LPS (1 μg/mL) for 2 h, and protein expression of p-JNK, JNK, p-ERK, ERK, p-p38, and p38 was determined by a western blotting assay. (**K**) RAW264.7 macrophages were pretreated with OE (20, 40, 80 μg/mL) for 3 h followed by stimulation with LPS (1 μg/mL) for 6 h. The IFN-β mRNA level was determined by quantitative real time PCR analysis. Data presented as Means ± SD. ** *p* < 0.01 vs. control group; # *p* < 0.05, ## *p* < 0.01 vs. LPS group; ns, no significance.

TLR4 regulates LPS-stimulated inflammation by forming a complex with its co-receptor MD2 on the cellular membrane [16]. Co-immunoprecipitation analysis found that LPS stimulation increased TLR4/MD2 complex formation, whereas OE significantly declined the quantity of MD2 bound to TLR4 in macrophages (Figure 3A,B). Further investigation revealed that OE simultaneously reduced LPS-induced generation of TLR4-associated MyD88 and TRIF, both of which are crucial adaptors in the TLR4 signaling pathway (Figure 3C–F). Additionally, OE suppressed the TLR4/MyD88-cascade-regulated MAPK activation, which is reflected by JNK, ERK, and p38 phosphorylation (Figure 3G–J). Similarly, the LPS-induced elevated mRNA level of TLR4/TRIF-cascade-regulated IFN-β was also reversed by OE (Figure 3K). Consequently, OE modulated both the MyD88 and the TRIF-dependent signaling pathways via inhibiting TLR4/MD2 complex formation in macrophages.

### 3.4. Silencing TLR4 Eliminated Anti-Inflammatory Effect of OE

**Figure 4:**
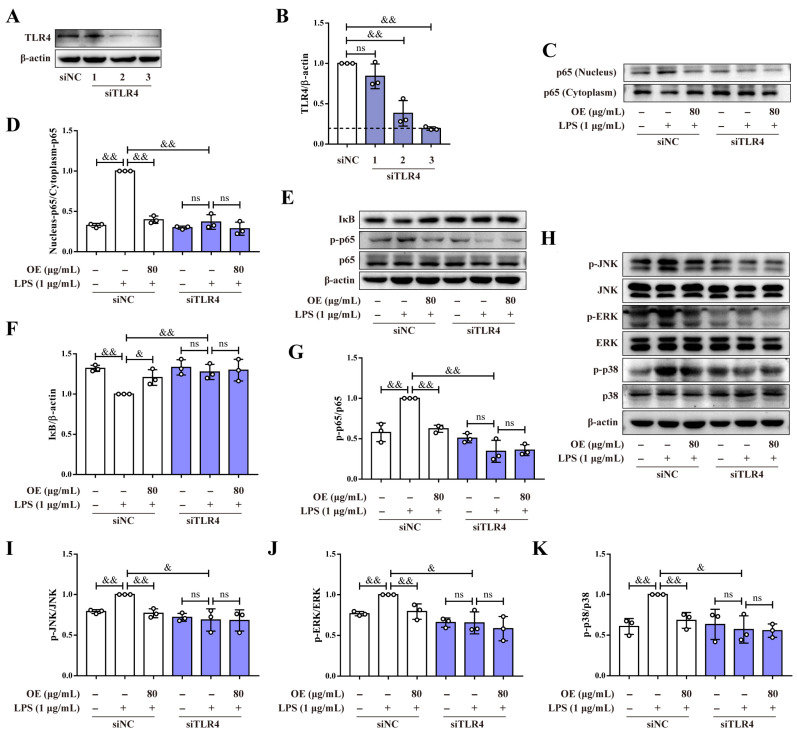
The anti-inflammatory effect of OE was counteracted by siTLR4. (**A**,**B**) RAW264.7 macrophages were transfected with siNC (100 nM) and siTLR4 (100 nM). The total protein expression of TLR4 was determined by a western blotting assay. (**C**–**K**) After transfecting siNC (100 nM) and siTLR4 (100 nM), RAW264.7 macrophages were pretreated with OE (80 μg/mL) for 3 h followed by stimulation with LPS (1 μg/mL) for 2 h. Nuclear and cytoplasmic protein expression of p65 (**C**,**D**) and the protein expression of IκB, p-p65, p65, p-JNK, JNK, p-ERK, ERK, p-p38, and p38 (**E**–**K**) were determined by a western blotting assay. Data presented as Means ± SD. & *p* < 0.05, && *p* < 0.01; ns, no significance.

To verify whether OE reversed LPS-stimulated inflammation through blocking TLR4/MD2 complex formation, siTLR4 was further studied. Western blotting displayed that NO.3 siTLR4 tremendously reduced TLR4 protein expression. Therefore, NO.3 siTLR4 was used for the following study (Figure 4A,B).

Data revealed that siTLR4 reversed both LPS-induced p65 nuclear translocation and phosphorylation, and degradation of IκB. Nevertheless, OE treatment did not show further reduction after siTLR4 interference in macrophages (Figure 4C–G). Similarly, the effect of OE on JNK, ERK, and p38 phosphorylation was neutralized by siTLR4 interference (Figure 4H–K). Together, OE mitigated inflammation by acting on TLR4.

### 3.5. OE Alleviated ROS Production by Suppressing NOX2 Endocytosis

**Figure 5:**
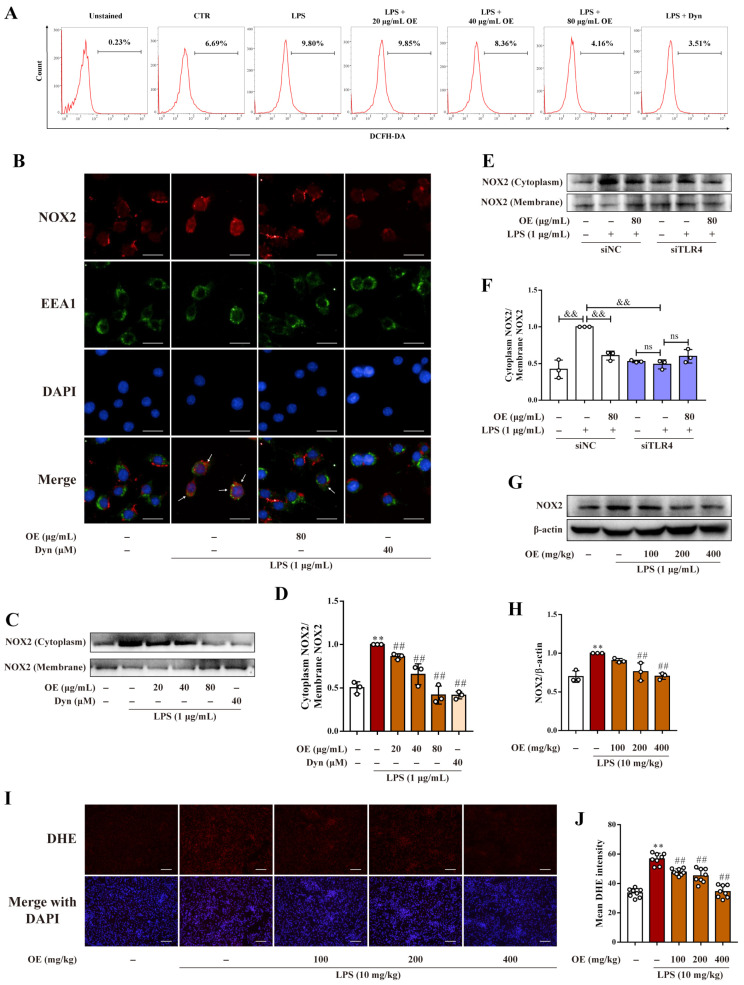
OE diminished ROS production by inhibiting NOX2 endocytosis. (**A**) RAW264.7 macrophages were pretreated with OE (20, 40, 80 μg/mL) for 3 h or with Dyn (40 μM) for 30 min followed by stimulation with LPS (1 μg/mL) for 6 h. ROS production was measured by a ROS detection kit via flow cytometry. (**B**–**D**) RAW264.7 macrophages were pretreated with OE (80 μg/mL) for 3 h or with Dynasore (40 μM) for 30 min followed by stimulation with LPS (1 μg/mL) for 4 h. Co-localization between NOX2 and EEA1 was determined by immunofluorescent staining (B). Scale bars: 20 μm. The cytoplasmic and membranal protein expression of NOX2 were determined by a western blotting assay (**C**,**D**). (**E**,**F**) After transfecting siNC (100 nM) or siTLR4 (100 nM), RAW264.7 macrophages were pretreated with OE (80 μg/mL) for 3 h followed by stimulation with LPS (1 μg/mL) for 4 h. Cytoplasmic and membranal protein expression of NOX2 were determined by a western blotting assay. (**G**–**J**) Mice were administered OE (100, 200, 400 mg/kg) by gavage for 7 days followed by intraperitoneal injection of LPS (10 mg/kg) and feeding for another 12 h. The total protein expression of NOX2 (**G**,**H**) and ROS production (**I**,**J**) in mice kidneys were determined by a western blotting assay and DHE staining, respectively. Scale bars: 50 μm. CTR: control. Data presented as Means ± SD. ** *p* < 0.01 vs. control group; ## *p* < 0.01 vs. LPS group; && *p* < 0.01; ns, no significance.

NOX2-mediated phagocytic ROS production is vital to initiate inflammation, and endocytosis appears to be a crucial part of NOX2 activation in phagocytes [7,17]. This study showed that OE, parallel to the endocytosis inhibitor Dynasore (Dyn), dose dependently reduced LPS-stimulated ROS production in RAW264.7 macrophages (Figure 5A). An immunofluorescence assay revealed that NOX2 showed a low co-localization level to the early endosome marker, early endosome antigen 1 (EEA1). However, LPS stimulation led to a pronounced elevation of co-localization between NOX2 and EEA1, whereas OE and Dynasore treatment reversed this phenomenon (Figure 5B). Meanwhile, LPS stimulation induced elevated protein expression of cytoplasmic NOX2. Conversely, this effect was restrained by OE and Dynasore treatment (Figure 5C,D). Intriguingly, silencing TLR4 counteracted the LPS-stimulated cytoplasmic translocation of NOX2, and OE did not show any further inhibition on NOX2 endocytosis (Figure 5E,F). Our results also showed that OE decreased the protein expression of NOX2 in mice kidneys (Figure 5G,H). Furthermore, DHE staining indicated that OE dose dependently diminished LPS-induced ROS generation in kidney tissues (Figure 5I,J). OE reversed LPS-induced NOX2 endocytosis and reduced ROS production in macrophages, which mitigated an inflammatory response by acting on the TLR4 signaling pathway.

### 3.6. OE Treatment Attenuated LPS-Induced AKI in Mice

**Figure 6:**
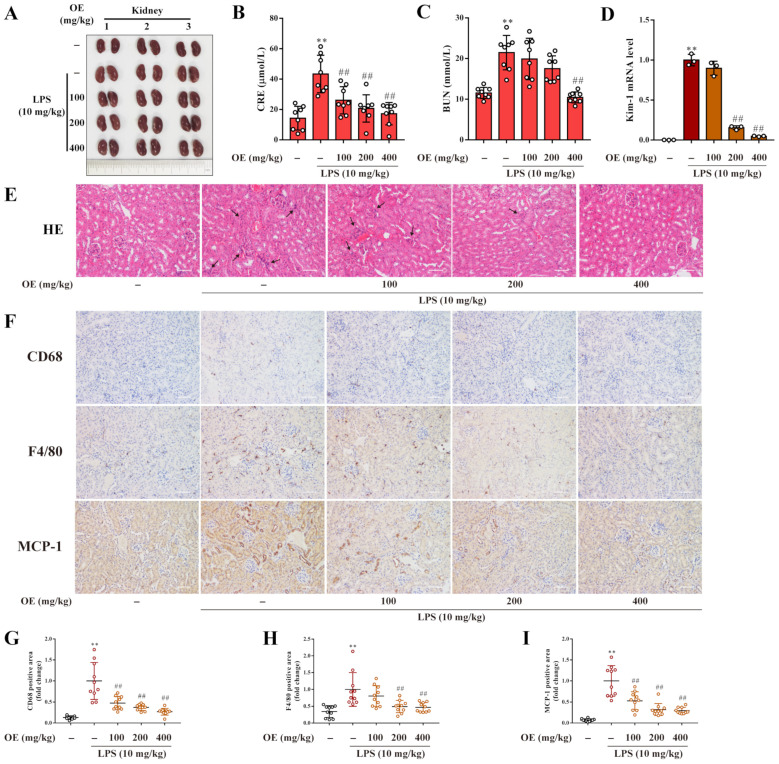
OE ameliorated LPS-induced AKI in mice. (**A**–**I**) Mice were administered OE (100, 200, 400 mg/kg) by gavage for 7 days followed by an intraperitoneal injection of LPS (10 mg/kg) and feeding for another 12 h. Kidney morphology (**A**) was observed. Serum CRE (**B**) and BUN (**C**) levels were measured by kits according to the manufacturer’s instructions. The mRNA level of Kim-1 in mice kidney was determined by quantitative real time PCR (**D**). Histopathology of kidneys was analyzed by HE staining (**E**). Arrows indicate the infiltration of inflammatory cells. Scale bars: 50 μm. Immunochemical analysis was applied to determine expression of CD68, F4/80, and MCP-1 in mice kidneys (**F**–**I**). Scale bars: 50 μm. Data presented as Means ± SD. ** *p* < 0.01 vs. control group; ## *p* < 0.01 vs. LPS group; ns, no significance.

LPS-induced AKI in mice was established to validate the in vivo pharmacological activity of OE. During the intragastric administration of OE, the body weight of the mice experienced no significant change compared to mice without administration (Figure S3). Morphologic analysis observed that kidneys in the LPS group presented as pale brown, whereas normal kidneys were dark red. After OE administration, the LPS-induced abnormal kidney appearance was restored to normal (Figure 6A). Biochemical analysis revealed that OE also ameliorated the LPS-induced high CRE and BUN levels in mice serum (Figure 6B,C). Kidney injury molecule-1 (Kim-1) is a transmembrane protein that is remarkably up-regulated after a series of kidney injuries [18]. Our results showed that OE markedly diminished the LPS-induced elevated mRNA level of Kim-1 in mice kidneys (Figure 6D). HE staining demonstrated that LPS stimulation led to the shedding of renal epithelial cells and the infiltration of inflammatory cells. Nonetheless, these phenomena were noticeably improved by OE treatment (Figure 6E). In addition, immunochemical analysis of the macrophage markers CD68 and F4/80, and chemoattractant cytokines MCP-1, were extensively distributed in the LPS-induced mice kidneys. However, OE distinctly decreased the expression of these inflammatory infiltration-associated cytokines (Figure 6F–I). Altogether, OE improved LPS-induced AKI in mice.

### 3.7. OE Diminished Inflammation in AKI Mice

**Figure 7:**
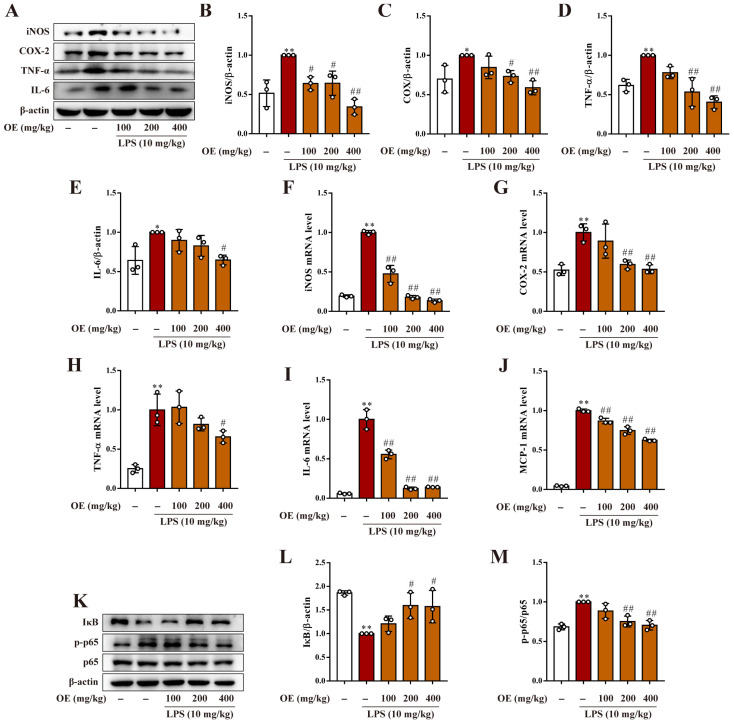
OE exhibited anti-inflammatory activity in AKI mice. (**A**–**M**) Mice were administered OE (100, 200, 400 mg/kg) by gavage for 7 days followed by the intraperitoneal injection of LPS (10 mg/kg) and feeding for another 12 h. The total protein expression of iNOS, COX-2, TNF-α, and IL-6 (**A**–**E**) in mice kidneys was determined by a western blotting assay. The mRNA level of iNOS (**F**), COX-2 (G), TNF-α (**H**), IL-6 (**I**), and MCP-1 (**J**) in mice kidneys was determined by quantitative real time PCR analysis. The protein expression level of IκB, p-p65, and p65 in mice kidneys was determined by a western blotting assay. Data presented as Means ± SD. * *p* < 0.05, ** *p* < 0.01 vs. control group; # *p* < 0.05, ## *p* < 0.01 vs. LPS group; ns, no significance.

We next explored the anti-inflammatory activity of OE in LPS-induced AKI mice. Results demonstrated that OE significantly decreased the enhanced protein expression of iNOS, COX-2, TNF-α, and IL-6 in AKI mice (Figure 7A–E). The LPS-induced high mRNA level of iNOS, COX-2, TNF-α, IL-6, and MCP-1 was reversed by OE (Figure 7F–J). Moreover, LPS-induced NF-κB activation, which is presented by IκB degradation and p65 phosphorylation, was reversed by OE (Figure 7K–M). Overall, in line with the in vitro study, OE reduced LPS-induced inflammation in AKI mice.

### 3.8. OE Inhibited TLR4/MD2 Complex Formation in AKI Mice

**Figure 8:**
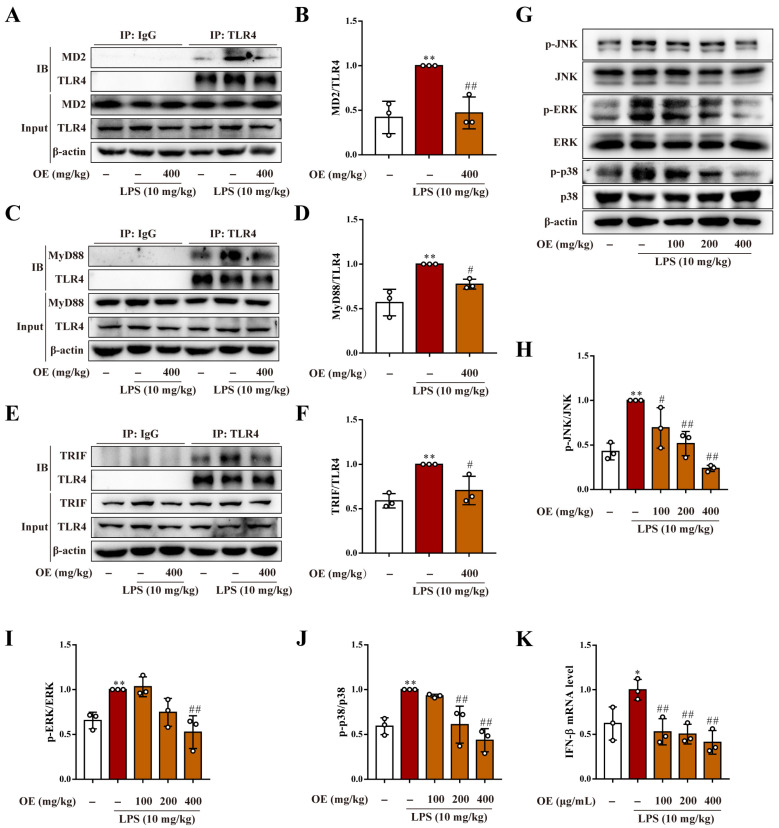
OE blocked TLR4/MD2 complex formation in AKI mice. (**A**–**K**) Mice were administered OE (100, 200, 400 mg/kg) by gavage for 7 days followed by the intraperitoneal injection of LPS (10 mg/kg) and feeding for another 12 h. TLR4/MD2 (**A**,**B**), TLR4/MyD88 (**C**,**D**), and TLR4/TRIF (**E**,**F**) complex formation in mice kidneys were determined by a co-immunoprecipitation assay. Protein expression of p-JNK, JNK, p-ERK, ERK, p-p38, and p38 (**G**–**J**) in mice kidneys was analyzed by a western blotting analysis. The mRNA level of IFN-β in mice kidney was determined by quantitative real time PCR analysis. Data presented as Means ± SD. * *p* < 0.05, ** *p* < 0.01 vs. control group; # *p* < 0.05, ## *p* < 0.01 vs. LPS group; ns, no significance.

The anti-inflammatory mechanism of OE was also investigated in AKI mice. Results revealed that OE vastly inhibited LPS-induced TLR4/MD2 complex formation (Figure 8A,B). Furthermore, OE decreased TLR4-bound MyD88 and TRIF adaptors, both of which were highly expressed in the kidney tissues of LPS-induced AKI mice (Figure 8C–F). In addition, OE also inhibited renal protein phosphorylation of JNK, ERK, and p38 in AKI mice (Figure 8G–J). Further, the LPS-induced high mRNA level of IFN-β was also reduced by OE treatment (Figure 8K). Taken together, OE exhibited anti-inflammatory activity by inhibiting TLR4/MD2 complex formation in LPS-induced AKI mice.

### 3.9. Analysis of Main Constituents in OE

The mzCloud matched 41 compounds in OE with a best match score greater than 85 points (Figure S4) (Table S3). There were 19 fatty acids, 13 sesquiterpenoids, 2 triterpenoids, and 7 other types of compounds among them.

## 4. Discussion

*O. himalaicus* has long been used to treat nephritis, rheumatoid arthritis, etc. [8,9,19]. Several studies reported that compounds from *O. himalaicus* showed anti-cancer and antibacterial activity via a variety of mechanisms [14,19,20,21,22]. However, few studies have been conducted to investigate the pharmacological activity surrounding the traditional medications of *O. himalaicus*. One study revealed that *O. himalaicus* facilitated lymphocyte proliferation with simultaneous anti-inflammatory and moderate antioxidant activity [23]. Additionally, after solvent extraction of the *O. himalaicus* ethanol extract by D101 macroporous resin, the aqueous phase and isolated compounds remarkably decreased the secretion of NO and several inflammatory cytokines in RAW264.7 macrophages [10]. These findings suggest that anti-inflammation is an inevitable explanation for the traditional medications of *O. himalaicus*. To systemically explore the anti-inflammatory activity of *O. himalaicus*, we screened out the most potent anti-inflammatory part of the *O. himalaicus* ethanol extract and investigated the mechanism by which it functioned. In our study, we found that D101 macroporous resin-eluted Fractions B and D showed powerful anti-inflammatory activity. Due to the significant cytotoxicity and low solid yield in Fraction D, only Fraction B, namely OE, was included in the next investigation. OE dose dependently alleviated LPS-stimulated inflammation in RAW264.7 macrophages. TLR4 is a conserved transmembrane protein that, unlike other TLRs, requires the co-receptor MD2 to function [24]. TLR4 was identified as an endotoxin receptor in the late 1990s, and it was found that TLR4 signaling pathway dysregulation contributes to a range of diseases [25]. Consequently, regulating the TLR4 signaling pathway is commonly considered a potential way to treat diseases. After LPS binds to the TLR4/MD2 complexes on the plasma membrane mediated by CD14, the TLR4/MD2 complexes undergo homodimerization to recruit TIRAP/MyD88, accounting for the encoding of inflammatory cytokines. The TLR4/MD2 complexes then internalize the endosome network to encode a type I interferon via TRAM/TRIF recruitment [5,6,26]. Targeting the TLR4 signaling pathway by various mechanisms, including blocking TLR4 dimerization or TLR4/MD2 complex formation, has been conducted extensively in the studies of natural products [27,28,29]. Our data revealed that OE exhibited an anti-inflammatory effect by inhibiting the TLR4/MD2 complex. Intriguingly, both adaptors of TLR4, MyD88, and TRIF were suppressed by OE. After TLR4 silencing, the activity of OE on LPS-induced inflammation was counteracted. Likewise, OE profoundly mitigated LPS-induced AKI and renal inflammation infiltration. These indicated that the TLR4/MD2 complex was involved in the anti-inflammatory activity of OE.

ROS are crucial components of the innate immune response that protects cells from external stimuli. It is recognized that phagocytes generate ROS primarily through the phagosome NADPH oxidase machinery, with NOX2 being the major one to govern ROS production [7,30]. Nonetheless, the over-production of ROS acts as a proinflammatory factor to aggravate local or systemic inflammation [31]. Endocytosis is a process in which cells engulf extracellular substances, including plasma membrane-located receptors of the cells with a cell membrane [32]. Studies demonstrated that palmitate induces the dynamin-dependent endocytosis of NOX2 and TLR4 to mediate ROS generation [33]. Additionally, inhibition of endocytosis by the Dynamin inhibitor Dynasore suppressed intracellular endotoxin-indued generation of ROS in neutrophils [17]. Moreover, hydroxychloroquine, an anti-malaria drug, was discovered to inhibit proinflammatory signaling by blocking NOX2 translocating to the endosome [34]. These investigations demonstrated the strong connection between NOX2-activated ROS production and endocytosis. Our results revealed that OE could decrease LPS-induced ROS production by restraining NOX2 endocytosis in macrophages. TLRs, particularly TLR4, have been shown to influence the ERK-p67phox-NOX2 axis in monocytes to regulate ROS generation [35]. Meanwhile, LPS-induced TLR4 activation modulated superoxide production by NOX2-dependent pathways in macrophages [36]. These studies revealed that TLR4 may participate in the regulation of NOX2 on ROS production. Our data found that silencing TLR4 vanished the effect of OE on NOX2 endocytosis. This indicated that the regulation of NOX2 endocytosis also contributed to OE blocking TLR4/MD2 complex formation.

Targeting the TLR4 signaling pathway is frequently considered a practical way to develop anti-inflammatory lead compounds [37,38]. Molecules that influence TLR4 activation are abundant in plenty of natural products [39]. *Glycyrrhiza uralensis*, ginger, and cinnamon bark are sources of molecules that block TLR4 dimerization [40,41,42]. Longanetin, isolated from Cornus fruits, is a novel TLR4 inhibitor to protect mice from rhabdomyolysis-induced acute kidney injury [43]. Beyond that, Curcumin derivatives, and naturally occurring chalcone derivatives, including the classical MD2 inhibitor, L6H21, have been revealed to target MD2 to restrict the TLR4 signaling pathway [44,45,46,47,48]. Our data showed that OE suppressed the MyD88 and TRIF-dependent cascades by blocking TLR4/MD2 complex formation, followed by reversing the LPS-induced activation of the NF-κB and MAPK signaling pathways, transcription of IFN-β, and ROS production regulated by NOX2-endocytosis. Thereafter, we tentatively identified 41 components from OE. Among these compounds, reynosin (PubChem CID 482788) was revealed to decrease inflammatory cytokine release. Carabrol (PubChem CID 15690483) and ursolic acid (PubChem CID 64945) were reported to exert an anti-inflammatory effect by inhibiting NF-κB activation [49,50]. (+/−)-Abscisic acid (PubChem CID 5375199) was found to suppress NLRP3 inflammasome and NF-κB activation to ameliorate inflammation [51,52]. Ilicic acid (PubChem CID 496073) was revealed to exert anti-inflammatory activity in vivo [53]. These compounds may partially account for the anti-inflammatory effect of OE. In sum, these data predominantly implied the potential to develop anti-inflammatory lead compounds from OE.

## 5. Conclusions

The fractional OE, obtained from *O. himalaicus*, significantly declined the LPS-induced expression of inflammation-associated genes and proteins but also restricted NOX2 endocytosis-regulated ROS production by inhibiting TLR4/MD2 complex formation. Additionally, OE could protect mice from AKI by withstanding inflammation. Our study provided the theoretical foundation of *O. himalaicus* on AKI. It also implied the possibility of developing anti-inflammatory lead compounds entailed in the TLR4 signaling pathway of *O. himalaicus*.

## Data Availability

The data presented in this study are available in this article and its supplementary materials.

## Supplementary Materials

The following supporting information can be downloaded at: https://www.mdpi.com/article/10.3390/antiox11122307/s1, Figure S1: Flow chart of the extraction and purification of OE; Figure S2: Cell viability of fraction A, fraction C, fraction D, and fraction E on RAW264.7 macrophages; Figure S3: Mice body weight change during OE administration; Figure S4: Component analysis in OE through UPLC-MS/MS. Table S1: Sequence (5′-3′) of siRNA; Table S2: Primer sequence (5′-3′) information; Table S3: Information of mzCloud-matched 41 components in OE.

## References

[1] Kellum J.A., Romagnani P., Ashuntantang G., Ronco C., Zarbock A., Anders H.-J. (2021). Acute kidney injury. Nat. Rev. Dis. Primers.

[2] Hoste E.A.J., Kellum J.A., Selby N.M., Zarbock A., Palevsky P.M., Bagshaw S.M., Goldstein S.L., Cerdá J., Chawla L.S. (2018). Global epidemiology and outcomes of acute kidney injury. Nat. Rev. Nephrol..

[3] Lauterbach M.A., Hanke J.E., Serefidou M., Mangan M.S.J., Kolbe C.C., Hess T., Rothe M., Kaiser R., Hoss F., Gehlen J. (2019). Toll-like Receptor Signaling Rewires Macrophage Metabolism and Promotes Histone Acetylation via ATP-Citrate Lyase. Immunity.

[4] Yamamoto M., Sato S., Hemmi H., Hoshino K., Kaisho T., Sanjo H., Takeuchi O., Sugiyama M., Okabe M., Takeda K. (2003). Role of adaptor TRIF in the MyD88-independent toll-like receptor signaling pathway. Science.

[5] Kagan J.C., Su T., Horng T., Chow A., Akira S., Medzhitov R. (2008). TRAM couples endocytosis of Toll-like receptor 4 to the induction of interferon-beta. Nat. Immunol..

[6] Ciesielska A., Matyjek M., Kwiatkowska K. (2021). TLR4 and CD14 trafficking and its influence on LPS-induced pro-inflammatory signaling. Cell. Mol. Life Sci..

[7] Bedard K., Krause K.H. (2007). The NOX family of ROS-generating NADPH oxidases: Physiology and pathophysiology. Physiol. Rev..

[8] Dimaer D.P. (1986). Jing Zhu Materia Medica.

[9] Pharmacopoeia Committee of the Ministry of Health (1995). Pharmaceutical Standards of the Ministry of Health of the People’s Republic of China. Tibet. Med..

[10] Qiu Y., Yang X., Xu J., Liu B., Li X. (2021). Benzofuran ε-caprolactam glucosides, amides and phenylpropanoid derivatives with anti-inflammatory activity from *Oxybaphus himalaicus*. Phytochemistry.

[11] Wang R., Dong Z., Zhang X., Mao J., Meng F., Lan X., Liao Z., Chen M. (2019). Evaluation of the Liver Toxicity of *Pterocephalus hookeri* Extract via Triggering Necrosis. Toxins.

[12] Mao J., Zhan H., Meng F., Wang G., Huang D., Liao Z., Chen M. (2022). Costunolide protects against alcohol-induced liver injury by regulating gut microbiota, oxidative stress and attenuating inflammation in vivo and in vitro. Phytother. Res..

[13] Mao J., Yi M., Wang R., Huang Y., Chen M. (2018). Protective Effects of Costunolide Against D-Galactosamine and Lipopolysaccharide-Induced Acute Liver Injury in Mice. Front. Pharmacol..

[14] Linghu L., Fan H., Hu Y., Zou Y., Yang P., Lan X., Liao Z., Chen M. (2014). Mirabijalone E: A novel rotenoid from *Mirabilis himalaica* inhibited A549 cell growth in vitro and in vivo. J. Ethnopharmacol..

[15] Yu H., Lin L., Zhang Z., Zhang H., Hu H. (2020). Targeting NF-κB pathway for the therapy of diseases: Mechanism and clinical study. Signal Transduct. Target. Ther..

[16] Su L., Athamna M., Wang Y., Wang J., Freudenberg M., Yue T., Wang J., Moresco E.M.Y., He H., Zor T. (2021). Sulfatides are endogenous ligands for the TLR4-MD-2 complex. Proc. Natl. Acad. Sci. USA.

[17] Lamb F.S., Hook J.S., Hilkin B.M., Huber J.N., Volk A.P., Moreland J.G. (2012). Endotoxin priming of neutrophils requires endocytosis and NADPH oxidase-dependent endosomal reactive oxygen species. J. Biol. Chem..

[18] Bonventre J.V. (2009). Kidney injury molecule-1 (KIM-1): A urinary biomarker and much more. Nephrol. Dial. Transplant..

[19] Long X., Meng F., Qu S., Ji H., Lan X., Chen M. (2022). Himalaflavone A-E, five new flavonoids from *Oxybaphus himalaicus*. Nat. Prod. Res..

[20] Lang L., Zhu S., Zhang H., Yang P., Fan H., Li S., Liao Z., Lan X., Cui H., Chen M. (2014). A natural phenylpropionate derivative from *Mirabilis himalaica* inhibits cell proliferation and induces apoptosis in HepG2 cells. Bioorg. Med. Chem. Lett..

[21] Li X., Yin M., Yang X., Yang G., Gao X. (2018). Flavonoids from *Mirabilis himalaica*. Fitoterapia.

[22] Zhou S.Y., Wang G.W., Zou Y.L., Deng L.Q., Liu M.X., Liao Z.H., Lan X.Z., Chen M. (2017). A new diphenyl ether derivative from *Mirabilis himalaica*. Nat. Prod. Res..

[23] Bo S., Dan M., Han W., Ochir S., Bao L., Liu L., Muschin T., Baigude H. (2022). Physicochemical properties, immunostimulatory and antioxidant activities of a novel polysaccharide isolated from *Mirabilis himalaica* (Edgew) Heim. RSC Adv..

[24] Park B.S., Song D.H., Kim H.M., Choi B.S., Lee H., Lee J.O. (2009). The structural basis of lipopolysaccharide recognition by the TLR4-MD-2 complex. Nature.

[25] Zhang Y., Liang X., Bao X., Xiao W., Chen G. (2022). Toll-like receptor 4 (TLR4) inhibitors: Current research and prospective. Eur. J. Med. Chem..

[26] Zanoni I., Ostuni R., Marek L.R., Barresi S., Barbalat R., Barton G.M., Granucci F., Kagan J.C. (2011). CD14 controls the LPS-induced endocytosis of Toll-like receptor 4. Cell.

[27] Yuan R., Huang L., Du L.J., Feng J.F., Li J., Luo Y.Y., Xu Q.M., Yang S.L., Gao H., Feng Y.L. (2019). Dihydrotanshinone exhibits an anti-inflammatory effect in vitro and in vivo through blocking TLR4 dimerization. Pharmacol. Res..

[28] Luo W., Yang L.B., Qian C.C., Ma B., Manjengwa G.M., Miao X.M., Wang J., Hu C.H., Jin B., Zhang L.X. (2021). Flavokawain B alleviates LPS-induced acute lung injury via targeting myeloid differentiation factor 2. Acta Pharmacol. Sin..

[29] Zhang Y., Xu T., Pan Z., Ge X., Sun C., Lu C., Chen H., Xiao Z., Zhang B., Dai Y. (2018). Shikonin inhibits myeloid differentiation protein 2 to prevent LPS-induced acute lung injury. Br. J. Pharmacol..

[30] Lambeth J.D. (2004). NOX enzymes and the biology of reactive oxygen. Nat. Rev. Immunol..

[31] Blaser H., Dostert C., Mak T.W., Brenner D. (2016). TNF and ROS Crosstalk in Inflammation. Trends Cell Biol..

[32] Sorkin A., von Zastrow M. (2009). Endocytosis and signalling: Intertwining molecular networks. Nat. Rev. Mol. Cell Biol..

[33] Kim S.Y., Jeong J.M., Kim S.J., Seo W., Kim M.H., Choi W.M., Yoo W., Lee J.H., Shim Y.R., Yi H.S. (2017). Pro-inflammatory hepatic macrophages generate ROS through NADPH oxidase 2 via endocytosis of monomeric TLR4-MD2 complex. Nat. Commun..

[34] Müller-Calleja N., Manukyan D., Canisius A., Strand D., Lackner K.J. (2017). Hydroxychloroquine inhibits proinflammatory signalling pathways by targeting endosomal NADPH oxidase. Ann. Rheum. Dis..

[35] Singh A., Singh V., Tiwari R.L., Chandra T., Kumar A., Dikshit M., Barthwal M.K. (2016). The IRAK-ERK-p67phox-Nox-2 axis mediates TLR4, 2-induced ROS production for IL-1β transcription and processing in monocytes. Cell. Mol. Immunol..

[36] Idelman G., Smith D.L.H., Zucker S.D. (2015). Bilirubin inhibits the up-regulation of inducible nitric oxide synthase by scavenging reactive oxygen species generated by the toll-like receptor 4-dependent activation of NADPH oxidase. Redox Biol..

[37] Wang Y., Zhang S., Li H., Wang H., Zhang T., Hutchinson M.R., Yin H., Wang X. (2020). Small-Molecule Modulators of Toll-like Receptors. Acc. Chem. Res..

[38] Li M., Wen J., Huang X., Nie Q., Wu X., Ma W., Nie S., Xie M. (2022). Interaction between polysaccharides and toll-like receptor 4: Primary structural role, immune balance perspective, and 3D interaction model hypothesis. Food Chem..

[39] Peri F., Calabrese V. (2014). Toll-like receptor 4 (TLR4) modulation by synthetic and natural compounds: An update. J. Med. Chem..

[40] Park S.J., Youn H.S. (2010). Suppression of homodimerization of toll-like receptor 4 by isoliquiritigenin. Phytochemistry.

[41] Ahn S.I., Lee J.K., Youn H.S. (2009). Inhibition of homodimerization of toll-like receptor 4 by 6-shogaol. Mol. Cells.

[42] Youn H.S., Lee J.K., Choi Y.J., Saitoh S.I., Miyake K., Hwang D.H., Lee J.Y. (2008). Cinnamaldehyde suppresses toll-like receptor 4 activation mediated through the inhibition of receptor oligomerization. Biochem. Pharmacol..

[43] Li J., Tan Y.J., Wang M.Z., Sun Y., Li G.Y., Wang Q.L., Yao J.C., Yue J., Liu Z., Zhang G.M. (2019). Loganetin protects against rhabdomyolysis-induced acute kidney injury by modulating the toll-like receptor 4 signalling pathway. Br. J. Pharmacol..

[44] Patel S.S., Acharya A., Ray R.S., Agrawal R., Raghuwanshi R., Jain P. (2020). Cellular and molecular mechanisms of curcumin in prevention and treatment of disease. Crit. Rev. Food Sci. Nutr..

[45] Gradisar H., Keber M.M., Pristovsek P., Jerala R. (2007). MD-2 as the target of curcumin in the inhibition of response to LPS. J. Leukoc. Biol..

[46] Zhang Y., Liu Z., Wu J., Bai B., Chen H., Xiao Z., Chen L., Zhao Y., Lum H., Wang Y. (2018). New MD2 inhibitors derived from curcumin with improved anti-inflammatory activity. Eur. J. Med. Chem..

[47] Zhang Y., Wu J., Ying S., Chen G., Wu B., Xu T., Liu Z., Liu X., Huang L., Shan X. (2016). Discovery of new MD2 inhibitor from chalcone derivatives with anti-inflammatory effects in LPS-induced acute lung injury. Sci. Rep..

[48] Kim S.Y., Koo J.E., Seo Y.J., Tyagi N., Jeong E., Choi J., Lim K.M., Park Z.Y., Lee J.Y. (2013). Suppression of Toll-like receptor 4 activation by caffeic acid phenethyl ester is mediated by interference of LPS binding to MD2. Br. J. Pharmacol..

[49] Lee H.J., Lim H.J., Lee D.Y., Jung H., Kim M.R., Moon D.C., Kim K.I., Lee M.S., Ryu J.H. (2010). Carabrol suppresses LPS-induced nitric oxide synthase expression by inactivation of p38 and JNK via inhibition of I-kappaBalpha degradation in RAW 264.7 cells. Biochem. Biophys. Res. Commun..

[50] Yuan L., Zhang L., Yao N., Wu L., Liu J., Liu F., Zhang H., Hu X., Xiong Y., Xia C. (2021). Upregulation of UGT1A1 expression by ursolic acid and oleanolic acid via the inhibition of the PKC/NF-κB signaling pathway. Phytomedicine.

[51] Chen X., Ding C., Liu W., Liu X., Zhao Y., Zheng Y., Dong L., Khatoon S., Hao M., Peng X. (2021). Abscisic acid ameliorates oxidative stress, inflammation, and apoptosis in thioacetamide-induced hepatic fibrosis by regulating the NF-кB signaling pathway in mice. Eur. J. Pharmacol..

[52] Zhao C.C., Xu J., Xie Q.M., Zhang H.Y., Fei G.H., Wu H.M. (2021). Abscisic acid suppresses the activation of NLRP3 inflammasome and oxidative stress in murine allergic airway inflammation. Phytother. Res..

[53] Máñez S., Recio M.C., Gil I., Gómez C., Giner R.M., Waterman P.G., Ríos J.L. (1999). A glycosyl analogue of diacylglycerol and other antiinflammatory constituents from *Inula viscosa*. J. Nat. Prod..

