# Supplementary material for: Oxybaphus himalaicus Mitigates Lipopolysaccharide-Induced Acute Kidney Injury by Inhibiting TLR4/MD2 Complex Formation
Source: Antioxidants (Basel). 2022 Nov 22;11(12):2307. doi: 10.3390/antiox11122307 (PMC9774781; doi:10.3390/antiox11122307)
Supplement: Supplementary file 1 [file antioxidants-11-02307-s001.zip › antioxidants-1993893-supplementary.pdf]

Oxybaphus himalaicus mitigates lipopolysaccharide-induced acute kidney injury by inhibiting TLR4/MD2 complex formation

Honghong Zhan [1], Qingxiu Pu [1], Xiaoliang Long [2], Wei Lu [1], Fancheng Meng [1], Guowei Wang [1], Zhihua Liao [2], Xiaozhong Lan [3], and Min Chen [1,*]

[1] Key Laboratory of Luminescence Analysis and Molecular Sensing (Southwest University), Ministry of Education; College of Pharmaceutical Sciences, Southwest University, Chongqing 400715, China

[2] School of Life Sciences, Integrative Science Center of Germplasm Creation in Western China (CHONGQING) Science City & Southwest University, The Provincial and Ministerial Co-founded Collaborative Innovation Center for R & D in Tibet Characteristic Agricultural and Animal Husbandry Resources, TAAHC-SWU Medicinal Plant Joint R&D Centre, Southwest University, Chongqing 400715, China

[3] TAAHC-SWU Medicinal Plant R&D Center, Tibet Agricultural and Animal Husbandry University, Nyingchi, Tibet 860000, China

* Correspondence: mminchen@swu.edu.cn

Table S1. Sequence (5’-3’) of siRNA

Table S2. Primer sequence (5’-3’) information

Table S3. Information of mzCloud-matched 41 components in OE.

Figure S1. Flow chart of the extraction and purification of OE.

Figure S2. Cell viability of fraction A (A), fraction C (B), fraction D (C) and fraction E (D) on RAW264.7 macrophages.

Figure S3. Mice body weight change during OE administration.

Figure S4. Component analysis in OE through UPLC-MS/MS.

Table S1. Sequence (5'-3') of siRNA

siRNA	Sense (5'-3')	Antisense (5'-3')	Accession
siTLR4#1	CUUCUUCAACCAAGAACAUTT	AUGUUCUUGGUUGAAGAAGTT	
siTLR4#2	CAAUUGACUUCAUUCAAGATT	UCUUGAAUGAAGUCAAUUGTT	NM_021297.3
siTLR4#3	CUAUCUAGAUCUUAGUAGATT	UCUACUAAGAUCUAGAUAGTT	

Table S2. Primer sequence (5'-3') information

Gene	Forward (5'-3')	Reverse (5'-3')	Accession
iNOS	GGCAGCCTGTGAGACCTTTG	GCATTGGAAGTGAAGCGTTTC	NM_010927.4
COX-2	TGAGTACCGCAAACGCTTCTC	TGGACGAGGTTTTTCCACCAG	NM_011198.5
TNF-α	ACTGAACTTCGGGGTGATCG	TGGTTTGTGAGTGTGAGGGTC	NM_001278601.1
IL-6	TCCAGTTGCCTTCTTGGGAC	GTGTAATTAAGCCTCCGACTTG	NM_031168.2
MCP-1	TGCCCTAAGGTCTTCAGCAC	AAGGCATCACAGTCCGAGTC	NM_011333.3
IFN-β	TCCACCAGCAGACAGTGTTTC	AGTTGAGGACATCTCCCACG	NM_010510.2
Kim-1	CCTTGTGAGCACCGTGGCTA	TGTTGTCTTCAGCTCGGGAATG	NM_001166632.1
GAPDH	CATGACCACAGTCCATGCCATCAC	TGAGGTCCACCACCCTGTTGCTGT	NM_001289726.2

Table S3. Information of mzCloud-matched 41 components in OE.

Compound	Name	Formula	Calculate molecular weight	Retention time (min)	PubChem CID	mzCloud Best match
1	Arachidic acid	$C_{20}H_{40}O_2$	312.30250	22.355	10467	99.4
2	(+/-)-Abscisic acid	$C_{15}H_{20}O_4$	264.13635	9.035	5375199	99.3
3	Ursolic acid	$C_{30}H_{48}O_3$	456.35979	20.306	64945	99.2
4	Linoleic acid	$C_{18}H_{32}O_2$	280.23994	20.598	5280450	99.1
5	Ilicic Acid	$C_{15}H_{24}O_3$	234.16170	16.450	496073	98.9
6	Petroselinic acid	$C_{18}H_{34}O_2$	282.25560	21.143	5282754	98.9
7	α-Eleostearic acid	$C_{18}H_{30}O_2$	278.22426	20.071	5281115	98.8
8	Ethyl myristate	$C_{16}H_{32}O_2$	256.23986	20.929	31283	98.8
9	Stearic acid	$C_{18}H_{36}O_2$	284.27131	21.690	5281	98.7
10	Pentadecanoic acid	$C_{15}H_{30}O_2$	242.22433	20.501	13849	98.7
11	Cis-7-Hexadecenoic acid	$C_{16}H_{30}O_2$	254.22418	20.345	5318393	98.7
12	Myristic acid	$C_{14}H_{28}O_2$	228.20871	20.012	11005	98.4
13	Lignoceric acid	$C_{24}H_{48}O_2$	368.36503	23.789	11197	98.3
14	3a,8-dihydroxy-3,5a,9-trimethyl-4,5,6,7,8,9b-hexahydro-3H-benzo[g][1]benzofuran-2-one	$C_{15}H_{22}O_4$	266.15201	14.287	14312974	98.3
15	5-hydroxy-4-methoxy-6,6,9a-trimethyl-4,5,5a,7,8,9-hexahydro-1H-benzo[e][2]benzofuran-3-one	$C_{16}H_{24}O_4$	302.14968	14.855	129008876	98.2
16	Docosanoic acid	$C_{22}H_{44}O_2$	340.33378	23.023	8215	98.2
17	Methyl 2-[4-ethenyl-2,6-dihydroxy-3-(3-	$C_{16}H_{24}O_5$	318.14440	10.958	45783164	98.2

	hydroxyprop-1-EN-2-YL)-4-methylcyclohexyl] prop-2-enoate					
18	Koninginin E	$C_{16}H_{26}O_4$	304.16515	15.108	11426065	98.0
19	(4E,6E)-2-(hydroxymethyl)-3-(3-methoxy-3-oxopropyl)deca-4,6-dienoic acid	$C_{15}H_{24}O_5$	306.14455	9.563	51136263	97.8
20	2-(2-hydroxybutyl)-1,3-dimethyl-4a,5,6,7,8,8a-hexahydro-2H-naphthalene-1-carboxylic acid	$C_{17}H_{28}O_3$	280.20413	16.538	85040117	97.6
21	(12Z)-9,10-Dihydroxyoctadec-12-enoic acid	$C_{18}H_{34}O_4$	296.23460	18.563	9966640	97.6
22	(E)-6-hydroxyoctadec-4-enoic acid	$C_{18}H_{34}O_3$	298.25035	18.916	51136456	97.3
23	Heptadecenoic acid	$C_{17}H_{32}O_2$	268.23985	20.813	5282747	97.3
24	[2-(Hydroxymethyl)-5,5,8a-trimethyl-1,4,4a,6,7,8-hexahydronaphthalen-1-yl]methanol	$C_{15}H_{26}O_2$	260.17549	17.302	13892459	97.3
25	4-(hydroxymethyl)-3,4a,8,8-tetramethyl-5,6,7,8a-tetrahydro-4H-naphthalen-1-one	$C_{15}H_{24}O_2$	236.17798	14.639	14489273	96.5
26	Hexadecanedioic acid	$C_{16}H_{30}O_4$	308.19661	17.929	10459	95.9
27	9-Hydroxy-5b,8,11a-trimethyl-1-prop-1-en-2-yl-1,2,3,4,5,6,7,7a,9,10,11,11b,12,13,13a,13b-hexadecahydrocyclopenta[a]chrysene-3a,5a,8-tricarboxylic acid	$C_{30}H_{44}O_7$	538.29119	15.460	45360106	95.9
28	Reynosin	$C_{15}H_{20}O_3$	230.13094	11.914	482788	93.7
29	2-[(2S,4aR,8aS)-2-hydroxy-4a-methyl-8-methylidene-3,4,5,6,7,8a-hexahydro-1H-naphthalen-2-	$C_{15}H_{22}O_3$	250.15657	12.372	23757244	93.2

	yl]prop-2-enoic acid					
30	[5,8a-bis(hydroxymethyl)-2,5-dimethyl-1,4,4a,6,7,8-hexahydronaphthalen-1-yl]methanol	$C_{15}H_{26}O_3$	276.17027	7.872	75528935	92.7
31	3,5-di-tert-Butyl-4-hydroxybenzyl alcohol	$C_{15}H_{24}O_2$	236.17735	18.211	6929	91.8
32	Acoric acid	$C_{15}H_{24}O_4$	290.14980	12.459	15558301	91.5
33	6-Hydroxyoctadec-4-enoic acid	$C_{18}H_{34}O_3$	320.23308	18.945	75614537	91.5
34	D-(+)-Maltose	$C_{12}H_{22}O_{11}$	364.09850	0.853	439186	90.5
35	12,13-DiHOME	$C_{18}H_{34}O_4$	296.23460	18.153	10236635	90.4
36	3-(4-Oxopentyl)-8-hydroxy-3,4-dihydro-1H-2-benzopyran-1-one	$C_{14}H_{16}O_4$	270.08702	7.476	23757076	90.2
37	2-[(2R)-4aalpha,8alpha-Dimethyl-8-hydroxydecahydronaphthalene-2alpha-yl]acrylic acid	$C_{15}H_{24}O_3$	274.15437	13.995	10901135	89.9
38	(9E,11Z)-8-hydroxyoctadeca-9,11-dienoic acid	$C_{18}H_{32}O_3$	318.21724	18.710	45359531	88.7
39	3,4-bis(hydroxymethyl)-4a,8,8-trimethyl-5,6,7,8a-tetrahydro-1H-naphthalene-1,4-diol	$C_{15}H_{26}O_4$	270.18259	13.243	23983651	87.8
40	Carabrol	$C_{15}H_{22}O_3$	250.15657	15.079	15690483	87.4
41	(3aS,5aS,9bR)-5a,9-dimethyl-3-methylidene-3a,4,6,7,8,9b-hexahydrobenzo[g][1]benzofuran-2,5-dione	$C_{15}H_{18}O_3$	246.12578	11.720	13855800	87.0

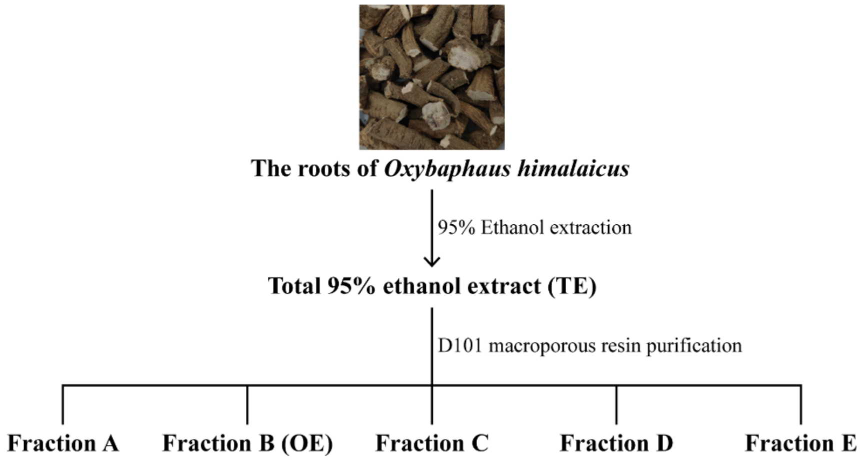

Figure S1. Flow chart of the extraction and purification of OE.

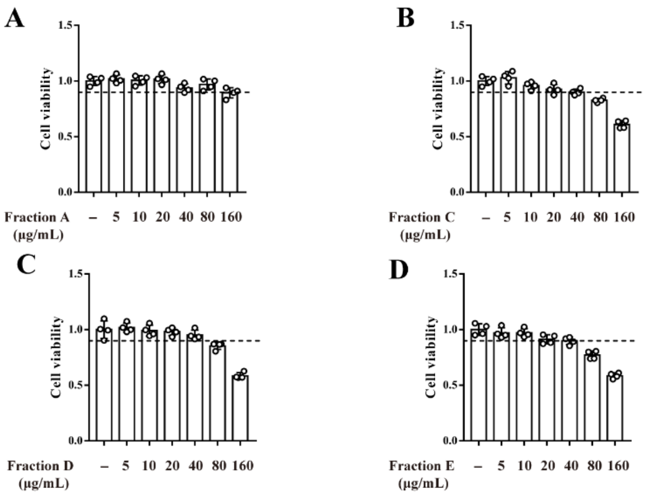

Figure S2. Cell viability of fraction A (A), fraction C (B), fraction D (C) and fraction E (D) on RAW264.7 macrophages.

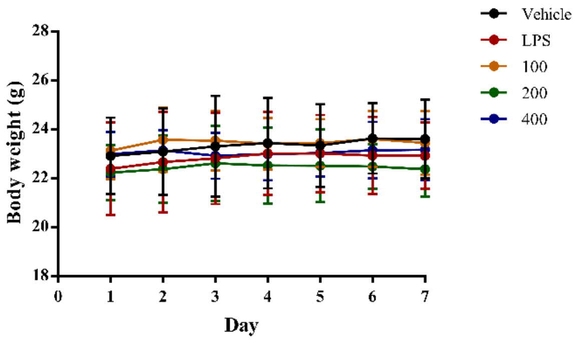

Figure S3. Mice body weight change during OE administration. Mice were administered OE (100, 200, 400 mg/kg) by gavage for 7 days, and body weight was measurement every day.

A

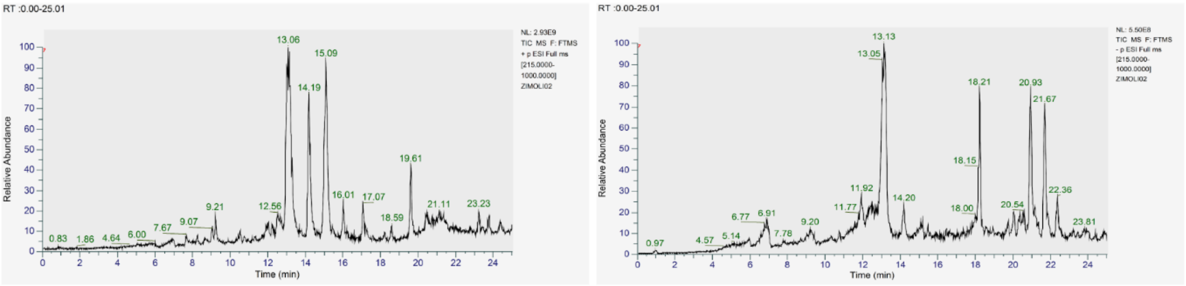

RT :0.00-25.01
Relative Abundance
Time (min)
NL: 2.93E9 TIC MS F: FTMS + p ESI Full ms [215.0000-1000.0000] ZIMOLI02
NL: 5.50E8 TIC MS F: FTMS - p ESI Full ms [215.0000-1000.0000] ZIMOLI02

B

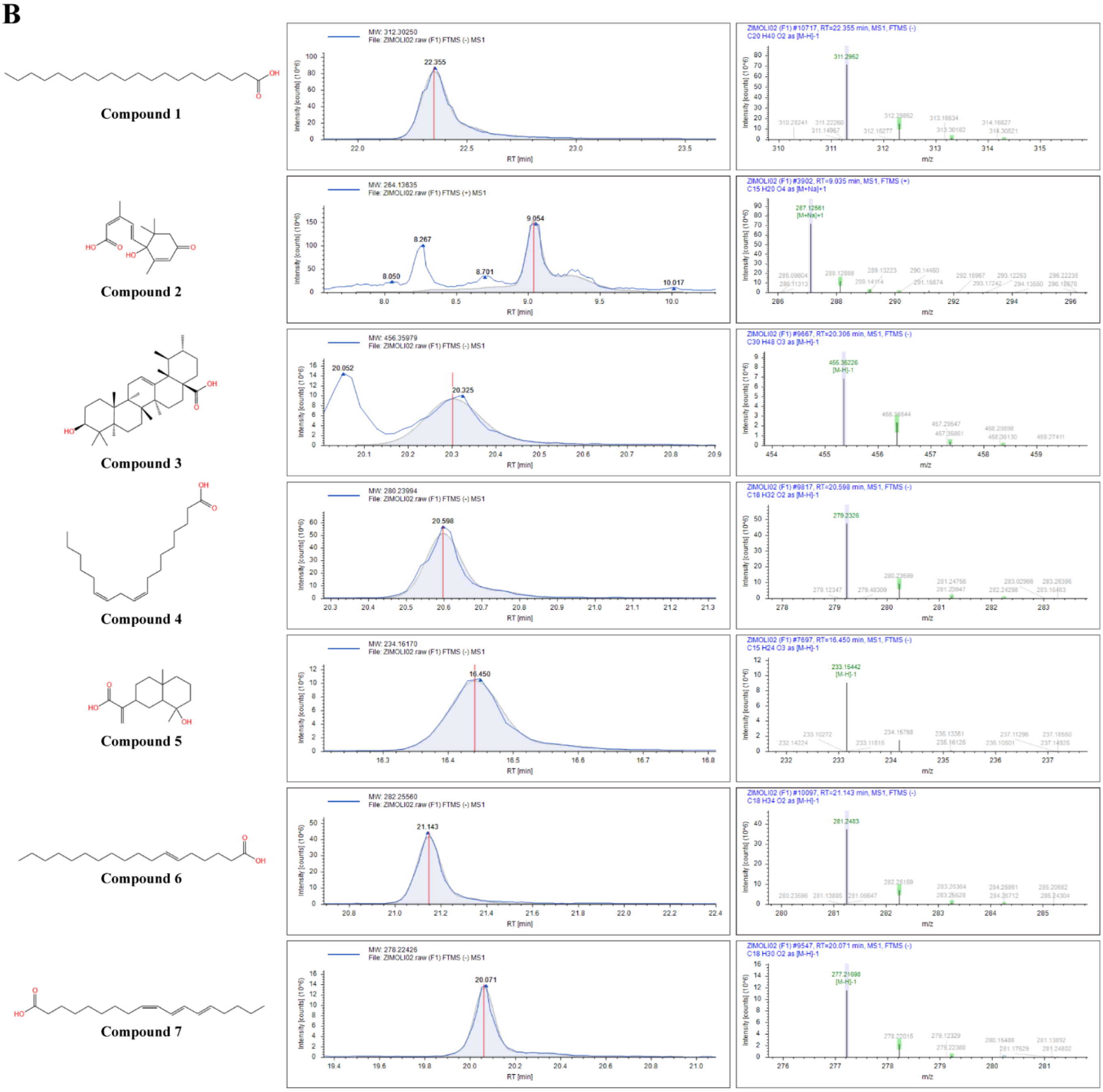

Compound 1
MW: 312.30250 File: ZIMOLI02.raw (F1) FTMS (-) MS1
ZIMOLI02 (F1) #10717, RT=22.355 min, MS1, FTMS (-) C20 H40 O2 as [M-H]-1
Compound 2
MW: 264.13635 File: ZIMOLI02.raw (F1) FTMS (+) MS1
ZIMOLI02 (F1) #3902, RT=9.035 min, MS1, FTMS (+) C15 H20 O4 as [M+Na]+1
Compound 3
MW: 456.35979 File: ZIMOLI02.raw (F1) FTMS (-) MS1
ZIMOLI02 (F1) #9667, RT=20.306 min, MS1, FTMS (-) C30 H48 O3 as [M-H]-1
Compound 4
MW: 280.23994 File: ZIMOLI02.raw (F1) FTMS (-) MS1
ZIMOLI02 (F1) #9817, RT=20.598 min, MS1, FTMS (-) C18 H32 O2 as [M-H]-1
Compound 5
MW: 234.16170 File: ZIMOLI02.raw (F1) FTMS (-) MS1
ZIMOLI02 (F1) #7697, RT=16.450 min, MS1, FTMS (-) C15 H24 O3 as [M-H]-1
Compound 6
MW: 282.25560 File: ZIMOLI02.raw (F1) FTMS (-) MS1
ZIMOLI02 (F1) #10097, RT=21.143 min, MS1, FTMS (-) C18 H34 O2 as [M-H]-1
Compound 7
MW: 278.22426 File: ZIMOLI02.raw (F1) FTMS (-) MS1
ZIMOLI02 (F1) #9547, RT=20.071 min, MS1, FTMS (-) C18 H30 O2 as [M-H]-1
RT [min]
m/z

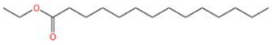

Compound 8

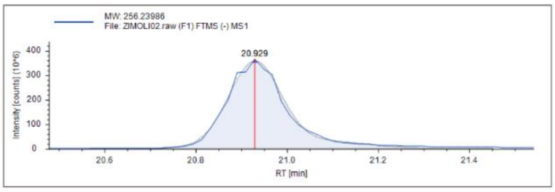

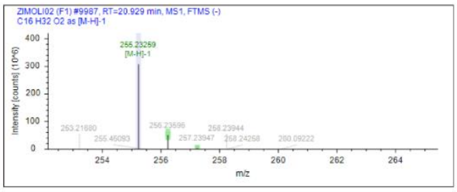

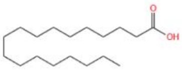

Compound 9

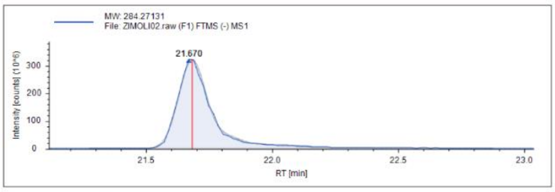

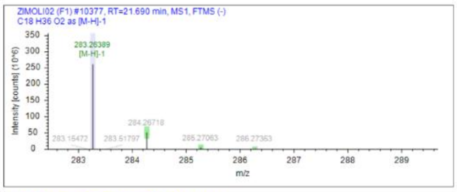

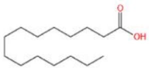

Compound 10

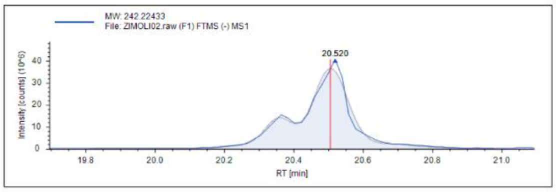

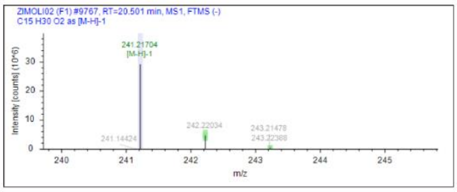

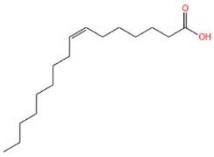

Compound 11

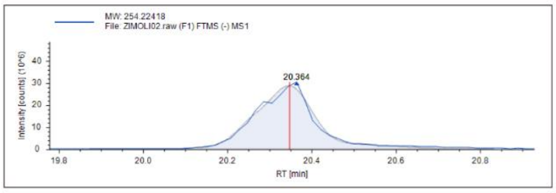

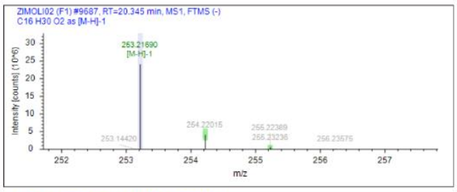

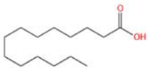

Compound 12

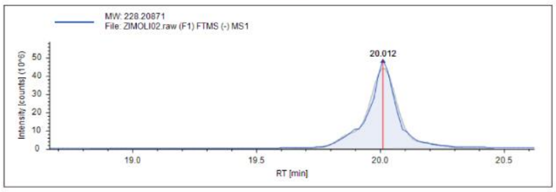

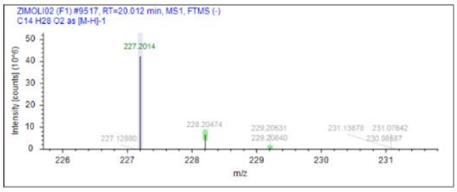

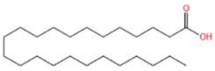

Compound 13

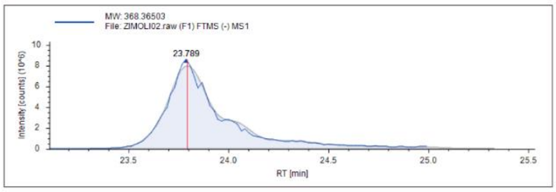

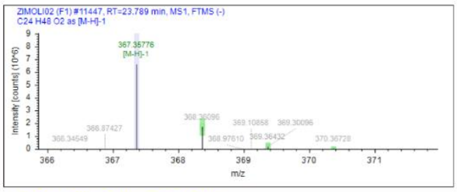

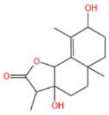

Compound 14

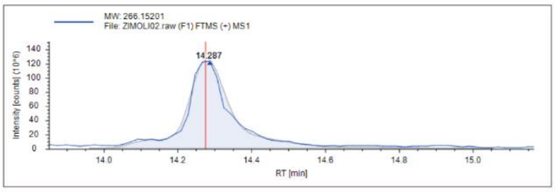

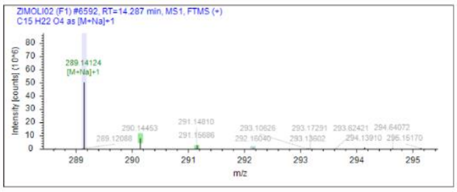

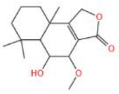

Compound 15

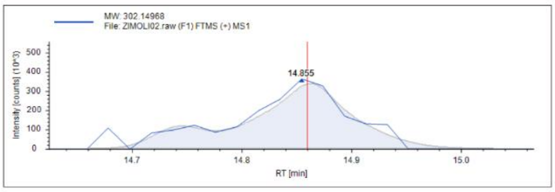

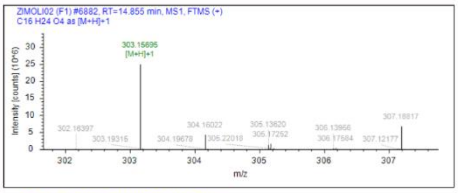

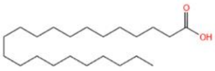

Compound 16

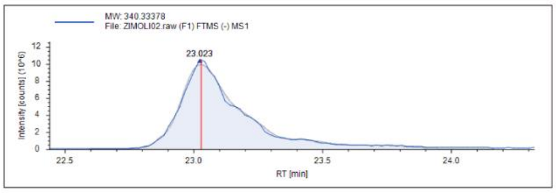

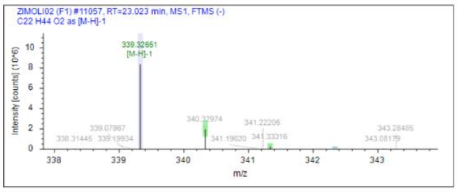

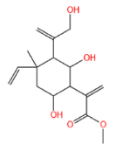

Compound 17

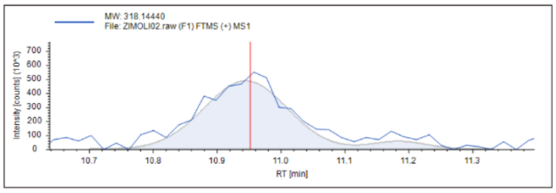

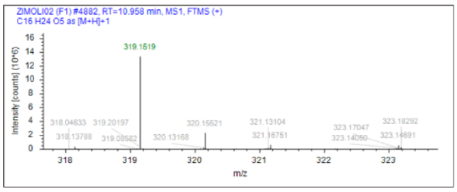

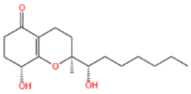

Compound 18

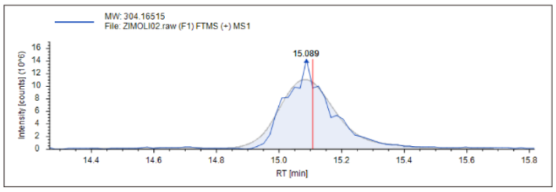

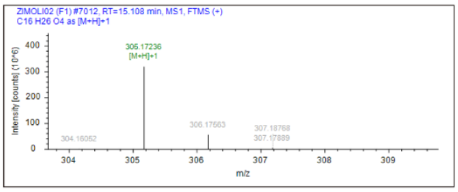

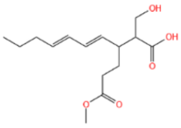

Compound 19

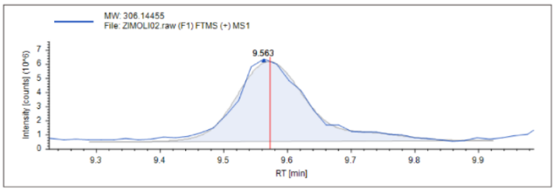

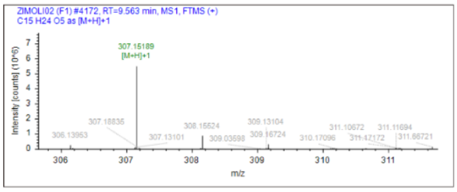

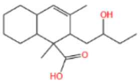

Compound 20

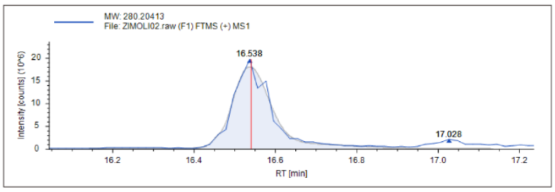

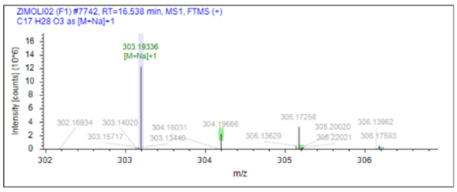

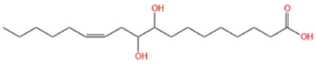

Compound 21

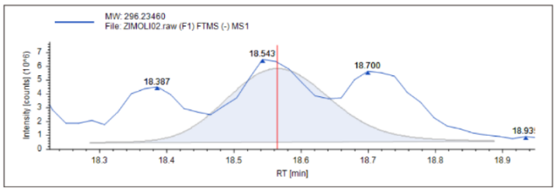

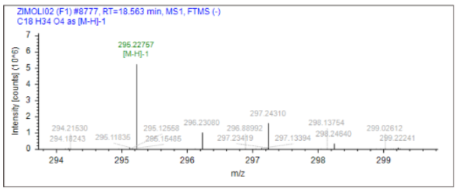

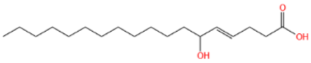

Compound 22

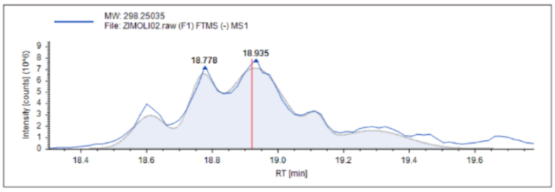

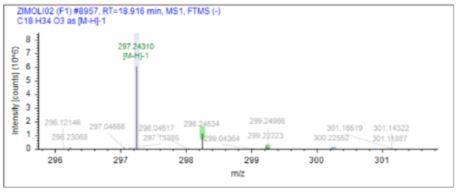

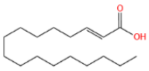

Compound 23

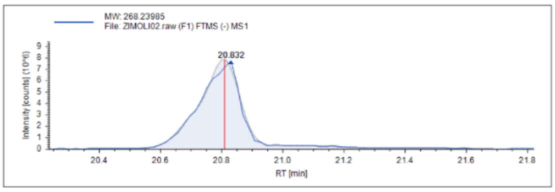

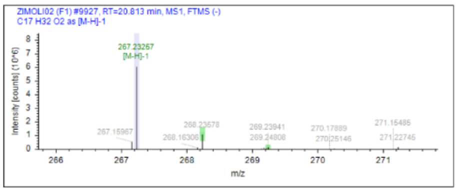

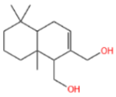

Compound 24

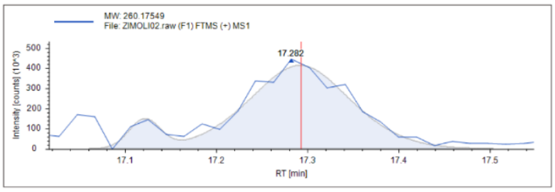

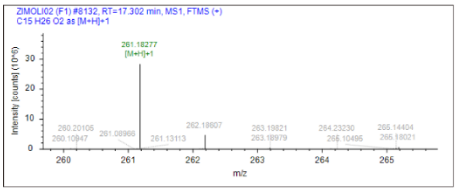

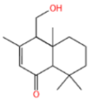

Compound 25

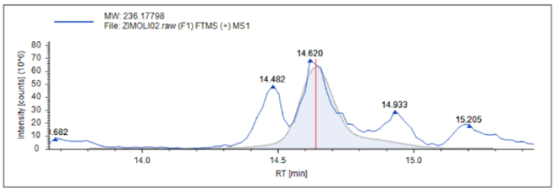

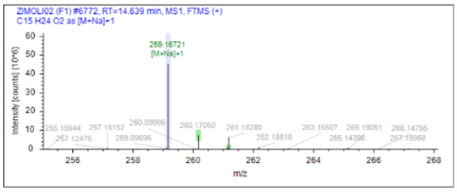

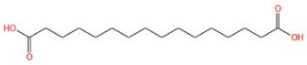

Compound 26

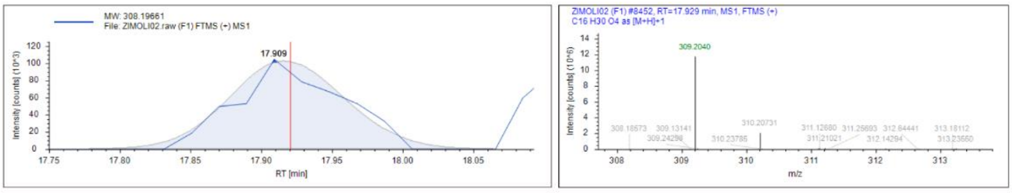

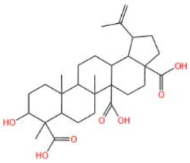

Compound 27

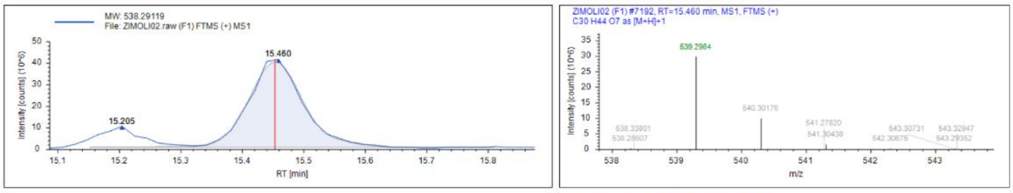

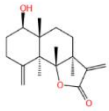

Compound 28

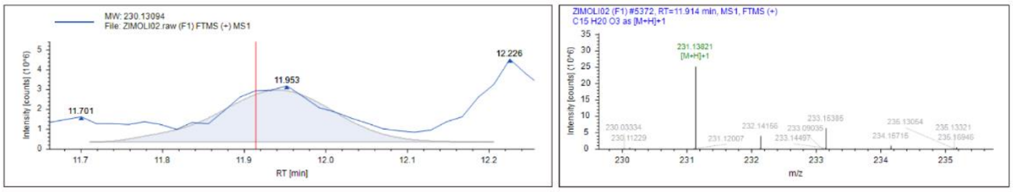

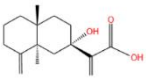

Compound 29

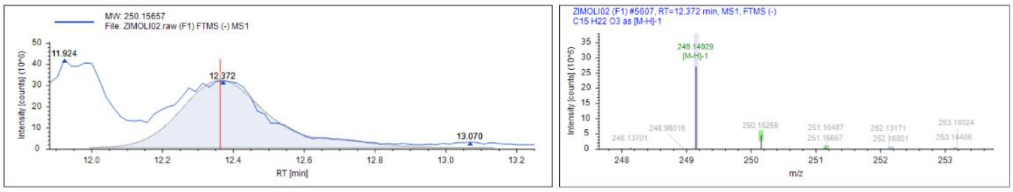

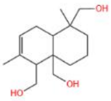

Compound 30

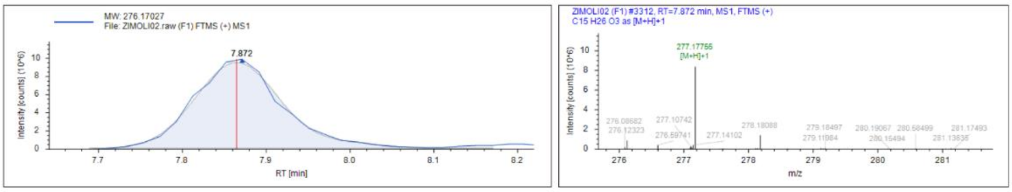

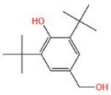

Compound 31

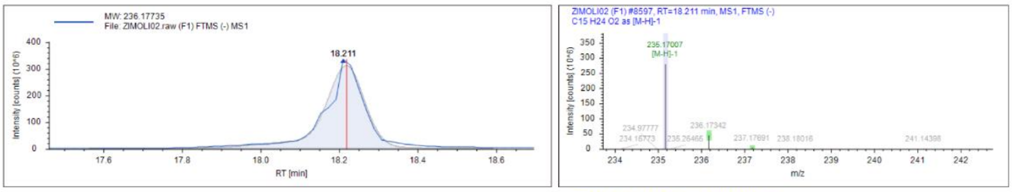

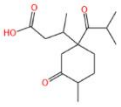

Compound 32

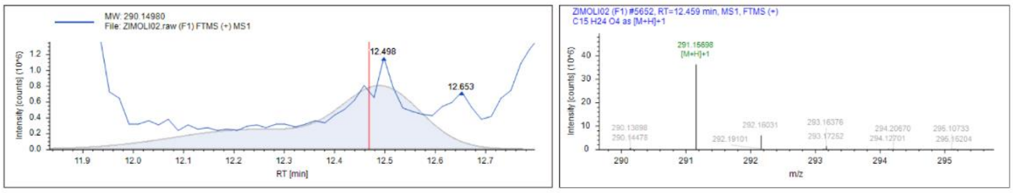

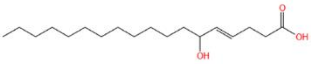

Compound 33

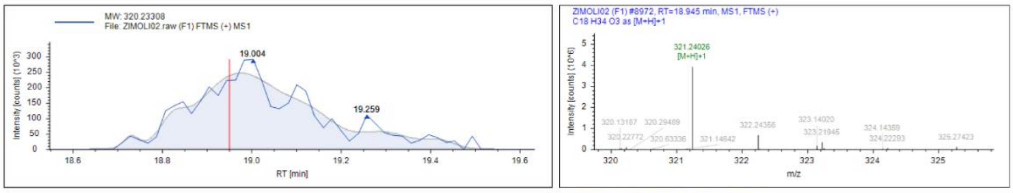

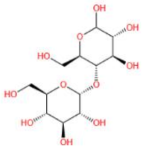

Compound 34

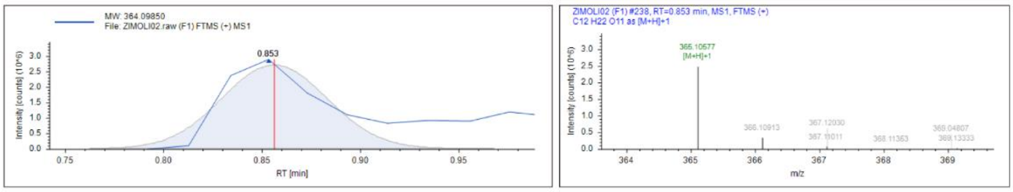

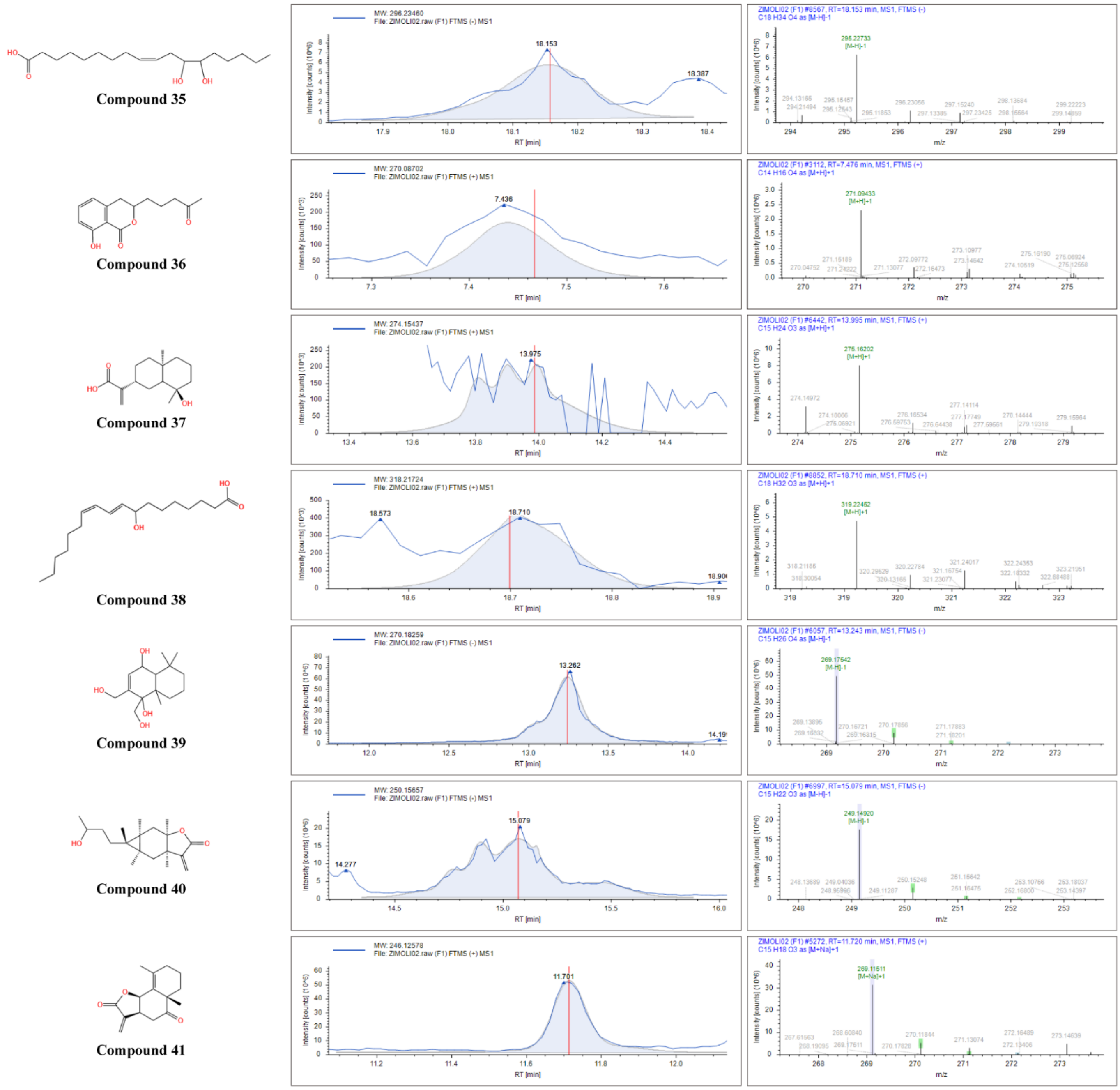

Figure S4. Component analysis in OE through UPLC-MS/MS. (A) Total ion chromatograms of OE. Left: positive mode. Right: negative mode. (B) Chemical structures, extracted ion chromatograms, and MS/MS images of 41 compounds tentatively identified in OE.
